# MLLT3 Regulates Melanoma Stemness and Progression by Inhibiting HMGB1 Nuclear Entry and MAGEA1 M^5^C Modification

**DOI:** 10.1002/advs.202408529

**Published:** 2024-12-24

**Authors:** Yaling Li, Hong Liu, Jingyi Li, Chang Fu, Bin Jiang, Bancheng Chen, Yanfen Zou, Bo Yu, Bing Song

**Affiliations:** ^1^ Institute of Biomedical and Health Engineering Shen Zhen Institutes of Advanced Technology Chinese Academy of Science Shenzhen Guangdong 518055 China; ^2^ Department of Dermatology Institute of Dermatology Peking University Shenzhen Hospital Shenzhen Peking University‐The Hong Kong University of Science and Technology Medical Center Shenzhen Guangdong 518036 China; ^3^ Department of Dermatology the First Hospital of China Medical University Shenyang Liaoning 110001 China; ^4^ Department of Otorhinolaryngology Xiang'an Hospital of Xiamen University Xiamen Fujian 361000 China

**Keywords:** m^5^C, MAPK, melanoma, MLLT3, P53, stemness

## Abstract

Melanoma stem cells are a kind of cells with self‐renewal and multi‐directional differentiation potential. They are one of the key factors in the occurrence, development and metastasis of melanoma. This study demonstrates that *MLLT3* is a transcription factor that regulates the stemness and progression of melanoma. *MLLT3* interacted with *HMGB1* to inhibit its entry into the nucleus, *MLLT3* interacted with *YBX1* to inhibit its reading of m^5^C of *MAGEA1*, thereby inhibiting the mRNA stability of *MAGEA1*, and directly transcribed P53 to inhibit the stemness, proliferation and metastasis of melanoma cells. This study further explored the potential mechanism of the interaction between miR‐542‐3p/miR‐3922‐3p and *MLLT3*. Furthermore, the scRNA‐seq of melanoma cells with *MLLT3* knock‐out resulted in important changes in cell subsets, activating the TP53 and MAPK pathways and transforming into stem cells. The results indicate that the transcription factor *MLLT3* is a suppressor gene that regulates the stemness and progression of melanoma, and is expected to become a target for melanoma therapy.

## Graphical abstract

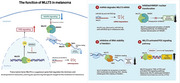

## 1 Introduction

Melanoma is the most malignant skin cancer, and its incidence has increased in the past few decades. It is estimated that more than 2 million people are diagnosed with melanoma every year.^[^ ^1^ ^]^ Melanoma is a heterogeneous cancer, which is composed of different cell subsets, including a small number of cancer stem cells and most non cancer stem cells.^[^ ^2^, ^3^ ^]^ In recent years, checkpoint immunotherapy has effectively prolonged the survival of patients with melanoma, but the recurrence and drug resistance caused by these melanoma stem cells are the key reasons for the death of patients with melanoma.^[^ ^4^ ^]^ Therefore, it is urgent to understand the regulatory network of melanoma stem cells.

Myeloid/Lymphoid Or Mixed‐Lineage Leukemia; Translocated To, 3 (*MLLT3*), also known as AF9, is a component of the superelongation complex (SEC), AF4/ENL/P‐TEFb complex (AEP) and DOT1L complex (DOTCOM). It promotes the key regulator of productive extension transcription and H3K79 methylation through phosphorylation of Pol II to maintain gene expression.^[^ ^5^, ^6^ ^]^ It is well known that *MLLT3* is a key regulator of human hematopoietic stem cell HSCs. Restoring the level of *MLLT3* in cultured human HSCs can protect the stemness and achieve in vitro expansion of transplantable HSCs.^[^ ^7^ ^]^ *MLLT3* also plays a regulatory role in different cancers. Depletion of AF9 can promote the proliferation, migration and glycolysis of colorectal cancer cells.^[^ ^6^ ^]^ For small cell lung cancer, inhibition of *MLLT3* can reduce the resistance of EGFR‐TKI induced by USP36 mutation to EGFR mutant NSCLC.^[^ ^8^ ^]^ For breast cancer, AF9 consumption significantly promotes the invasion and migration of breast cancer cells.^[^ ^9^ ^]^ For hepatocellular carcinoma, down‐regulation of *MLLT3* can reduce the proliferation of hepatocellular carcinoma cells and block the formation of solid tumors.^[^ ^10^ ^]^ For oral squamous cell carcinoma, knockdown of *MLLT3* can reduce the migration and invasion of oral squamous cell carcinoma cells.^[^ ^11^ ^]^ However, the effect of *MLLT3* on melanoma progression has not been confirmed.

MAGE family member A1 (*MAGEA1*) is a member of MAGEA gene family and has tumor promoting activity in melanoma, which may be due to the activation of P‐C‐JUN or ERK‐MAPK signaling pathway.^[^ ^12^ ^]^ Studies have shown that *MAGEA1* can inhibit the transcription of p53 in medulloblastoma and esophageal squamous cell carcinoma.^[^ ^13^, ^14^ ^]^ However, whether *MAGEA1* can regulate p53 transcriptional function in melanoma remains unknown. High mobility group box 1 (*HMGB1*) is a non histone chromatin related protein widely distributed in eukaryotic cells, which can be transferred to the extracellular environment as a risk warning protein, activate the immune response, and participate in the regulation of inflammation and cancer progression.^[^ ^15^ ^]^ In melanoma, *HMGB1* can be secreted through p53, NF‐κB stimulates melanoma proliferation, metastasis and increased drug resistance.^[^ ^16^ ^]^ However, whether *HMGB1* can regulate the stemness of melanoma cells through *MLLT3* interaction remains unknown. Y‐box binding protein 1 (YBX1) is an RNA/DNA binding multifunctional protein. It is a recognized carcinogenic transcription factor, which can regulate cell apoptosis, translation, cell proliferation, mRNA splicing, repair, differentiation and stress response.^[^ ^17^ ^]^ 5‐methylcytosine (m^5^C) is recognized as a regulator of gene expression and genomic stability. As a reader of m^5^C, YBX1 plays an important role in the occurrence and development of tumors.^[^ ^18^ ^]^ Some studies suggest that silencing YBX1 expression significantly inhibits the stemness of melanoma stem cells,^[^ ^19^ ^]^ and *NSUN2* regulates the proliferation and migration of uveal melanoma cells by mediating RNA m^5^C modification.^[^ ^20^ ^]^ However, how *MLLT3* regulates m^5^C modification remains unclear.

This study further clarified the important role of *MLLT3* in melanoma and explored its potential molecular mechanism, aiming to provide a new treatment strategy for melanoma.

## 2 Results

### 2.1 Identification of *MLLT3* as a Suppressor Gene of Melanoma

**Figure 1:**
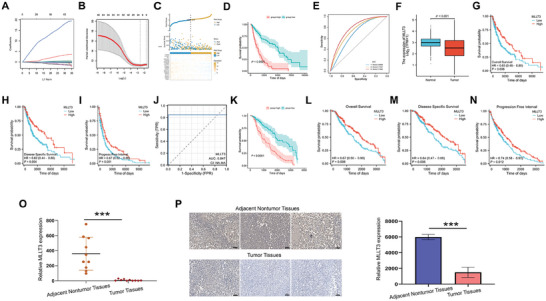
*MLLT3* was identified as a tumor suppressor gene of melanoma. A) The change track of each independent variable. The horizontal axis represents the log value of the independent variable lambda, and the vertical axis represents the coefficient of the independent variable. B) The confidence interval under each lambda. C) The distribution map of the training set risk scoring model. D) The survival curve of the training set risk score model. E) ROC curve of training set risk scoring model. F) The expression of *MLLT3* in TCGA‐SKCM was low in melanoma. G) The OS of melanoma patients with low expression of *MLLT3* in TCGA‐SKCM was poor. H) The DSS of melanoma patients with low expression of *MLLT3* in TCGA‐SKCM was poor. I) PFI of melanoma patients with low expression of *MLLT3* in TCGA‐SKCM was poor. J) *MLLT3* predicts the ROC curve of melanoma. K) The survival curve of the training set risk score model (within 10 years). L) The OS of melanoma patients with low expression of MLLT3 in TCGA‐SKCM was poor (within 10 years). M) The DSS of melanoma patients with low expression of MLLT3 in TCGA‐SKCM was poor (within 10 years). N) The PFI of melanoma patients with low expression of MLLT3 in TCGA‐SKCM was poor (within 10 years). O) PCR expression in melanoma tissue. P) Immunohistochemical results of melanoma tissue. ***P < 0.001.

In order to identify the genes related to melanoma stem cell, we first constructed a risk score model of melanoma stemness related genes based on the expression profile data of SKCM in TCGA database and cancer stemness related genes. In the TCGA‐SKCM training set, 398 candidate genes were identified by univariate Cox analysis, and 64 genes were retained (Table S2, Supporting Information). Next, Lasso was used to further screen the variables, and finally 18 genes were retained (**Figure** **1**A,B). Based on the above 18 genes, the risk score model of tumor stem cell genes was constructed by multivariate Cox regression. By dividing the samples into high‐risk group and low‐risk group, it can be observed that the proportion of death samples in the high‐risk group is higher (Figure 1C), and the OS (overall survival) of patients in the high‐risk score group is significantly lower than that in the low‐risk score group (Figure 1D). And this model has a good ability to predict the survival time, and its AUC of 1, 3, and 5 years are 0.6868, 0.7427, and 0.7862 respectively (Figure 1E). Next, we use the test set, the overall set, GSE19234 and GSE65904 data sets to verify the model, and the results show that the model has good prediction ability (Figure S1A–L, Supporting Information). Among the 18 genes in this model, *MLLT3* is a novel melanoma tumor suppressor gene, which is the object of our further study. In TCGA‐SKCM, the expression of *MLLT3* was low in melanoma tissues (Figure 1F), and the OS, DSS (disease specific survival) and PFI (progression free interval) of melanoma patients with low expression of *MLLT3* were poor (Figure 1G–I). In addition, *MLLT3* has good predictive ability in TCGA‐SKCM, and the AUC value reaches 0.847 (Figure 1J). To investigate the impact of MLLT3 on the prognosis of melanoma patients within a medium to short‐term period (within 10 years), we subsequently re‐analyzed the prognosis of high‐risk and low‐risk score groups, as well as the prognostic role of MLLT3 in melanoma patients. The results still revealed that the OS of patients in the high‐risk score group is significantly lower than that in the low‐risk score group (Figure 1K), and the OS, DSS and PFI of melanoma patients with low expression of MLLT3 were poor (Figure 1L–N). PCR and immunohistochemistry results also confirmed that MLLT3 was low expressed in melanoma tissues (Figure 1O,P). In conclusion, our results show that low expression of *MLLT3* plays an important role in promoting malignant progression of melanoma, and is closely related to poor prognosis.

### 2.2 CRISPR/Cas9‐Mediated *MLLT3* Knockout (KO) Promoted Proliferation, Metastasis, Invasion, and Stemness in Melanoma Cells

**Figure 2:**
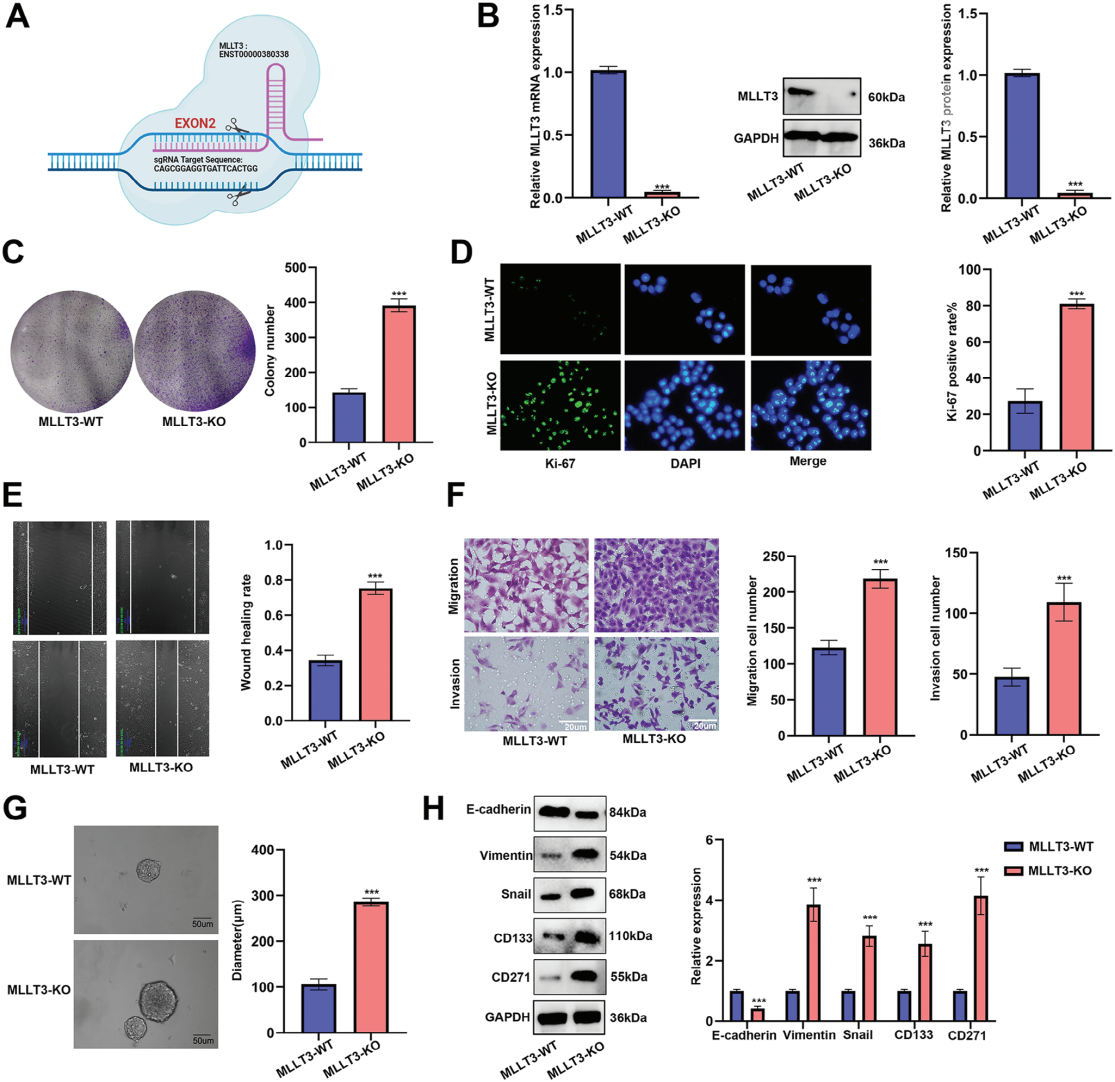
CRISPR/Cas9‐mediated *MLLT3* knockout (KO) promoted proliferation, metastasis, invasion, and stemness of melanoma cells. A) Schematic model of the *MLLT3* knockout CRISPR/Cas9 system. B) The expression of *MLLT3* was measured by qRT‐PCR and western blot after knockout. C,D) The effect of *MLLT3* on cell proliferation was detected by colony formation and Ki‐67 immunofluorescence staining after *MLLT3* knockout. E,F) Wound healing and transwell assays were performed to detect the effect of *MLLT3* on cell metastasis and invasion. G) The effects of *MLLT3* knockout on sphere‐forming ability were detected by sphere‐forming assays. H) The protein levels of EMT and stemness markers were measured by western blot assay. ***P < 0.001.

To determine the functional significance of *MLLT3* in melanoma, CRISPR/Cas9‐mediated knockout system of *MLLT3* and *MLLT3* overexpression cDNA vector were constructed (**Figure** **2**A). As shown in Figure S2A (Supporting Information), we also detected the expression of *MLLT3* in PIG‐1 cell and several types of melanoma cells including A375, HT‐144, WM‐115 and SK‐MEL‐2 by using qRT‐PCR. Of these cells, A375 cells with the lowest expression of *MLLT3* were transfected with a *MLLT3* cDNA vector, and SK‐MEL‐2 cells with the highest expression level of *MLLT3* were transfected with a CRISPR/Cas9‐mediated knockout system. As shown in Figure 2B, we achieved significant *MLLT3* protein KO in our selected clones. Next, the effects of *MLLT3* KO and overexpression on proliferative potential of melanoma cells were assessed by colony formation assay and Ki67 immunofluorescent staining. As shown in Figure 2C,D and Figure S2B (Supporting Information), *MLLT3* KO resulted in a marked increase in colony formation and Ki67 staining, while overexpression of *MLLT3* significantly decreased the colony formation of A375 cells. Next, we detected the effect of *MLLT3* on cell metastasis by wound healing and transwell assays. As shown in Figure 2E,F and Figure S2C (Supporting Information), *MLLT3* KO enhanced metastasis and invasion in SK‐MEL‐2 cells, and conversely, *MLLT3* overexpression remarkably inhibited cell migration and invasion in A375 cells. In addition, we found that knockout of *MLLT3* increased the sphereforming ability of melanoma cells (Figure 2G). Moreover, we further detected *MLLT3* expression on melanoma cell epithelial‐mesenchymal transition (EMT) and stemness by western blot assay. As shown in Figure 2H, *MLLT3* KO cells expressed a low level of E‐cadherin and high levels of Vimentin, Snail (EMT markers), and CD133 (stemness marker) compared with *MLLT3* WT cells, while overexpression of *MLLT3* inhibited the expression of EMT markers and stemness mark (Figure S2D, Supporting Information). Based on *MLLT3* as the variable for grouping, followed by GO (Gene Ontology) analysis, it is demonstrated that *MLLT3* is associated with the stem cell proliferation, differentiation, development and population maintenance (Figure S2E, Supporting Information). In addition, to reveal the role of *MLLT3* in melanoma stem cells, the expression of stemness‐related genes were evaluated in melanoma stem cells after *MLLT3* overexpression or knockout. As shown in Figure S2F (Supporting Information), *MLLT3* overexpression significantly reduced the expression of stemness factors of A375 derived stem cells, while knockout of *MLLT3* promoted these stemness factors. To sum up, our results certified that *MLLT3* inhibited proliferation, metastasis, invasion, and stemness in melanoma cells.

### 2.3 *MLLT3* was Regulated by miR‐542‐3p and miR‐3922‐3p in Melanoma

**Figure 3:**
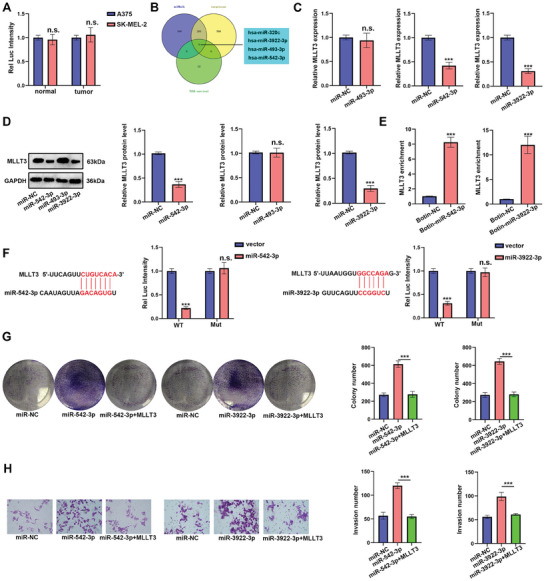
*MLLT3* was regulated by miR‐542‐3p and miR‐3922‐3p in melanoma. A) Dual‐luciferase reporter assay was performed to test the *MLLT3* promoter activity. B) TCGA survival analysis, miRwalk and Targetscan were used to predict the potential miRNA, which could target 3′UTR of homo sapiens *MLLT3*. C,D) The mRNA and protein levels of *MLLT3* was measured by qRT‐PCR after miRNA mimics transfection. E) The interaction between *MLLT3* mRNA and miR‐542‐3p/miR‐3922‐3p was detected by RNA pull‐down assay. F) Dual‐luciferase reporter assay was performed to test the activity of wild type or mutant 3′UTR of *MLLT3* by co‐transfection with miR‐542‐3p/miR‐3922‐3p. G) Cell proliferation of A375 cells was measured by colony formation after miRNAs and *MLLT3* co‐transfection. H) Cell invasion of A375 cells was measured by transwell assay after miRNAs and *MLLT3* co‐transfection. n.s. *P* > 0.05, ***P < 0.001.

To identify the upstream molecular of *MLLT3*, we examined the promoter activity of *MLLT3* gene by introducing its promoter from a tumor or normal tissue into pGL3B and found that the promoter activity of the *MLLT3* gene in tumor or normal tissue has no difference, indicating the expression of *MLLT3* was regulated in a post‐transcription manner in melanoma (**Figure** **3**A). It is well‐known that miRNAs regulated sat least 30% of mRNA mature and played a pivotal role in cancer. We further predicted the potential miRNAs that could target *MLLT3* mRNA to figure out how *MLLT3* was silenced in melanoma. As shown in Figure 3B, four miRNAs predicted by miRWalk and TargetScan database may be involved in regulating *MLLT3* mRNA. We did not detect miR‐320c in melanoma tissues and excluded it, we next measured the effect of miR‐493‐3p, miR‐542‐3p and miR‐3922‐3p on *MLLT3* expression by qRT‐PCR and western blot assays. As shown in Figure 3C,D, overexpression of miR‐524‐3p and miR‐3922‐3p significantly decreased the mRNA and protein levels of MLLT3. By using TCGA database, we also identified that miR‐542‐3p and miR‐3922‐3p was up‐regulated in melanoma and negatively correlated with overall survival rate (Figure S3A–C, Supporting Information). Next, the interaction between *MLLT3* and miR‐542‐3p/miR‐3922‐3p was confirmed by RNA pull‐down assay (Figure 3E). In addition, the interaction region of *MLLT3* and miR‐542‐3p/miR‐3922‐3p was predicted by TargetScan database and then constructed into Luciferase Report vectors. As shown in Figure 3F, after mutating, miR‐542‐3p and miR‐3922‐3p could not affect *MLLT3* 3′UTR any more, proving that miR‐542‐3p and miR‐3922‐3p regulated *MLLT3* mRNA stability by targeting its 3′UTR. In order to investigate the effect of miR‐542‐3p and miR‐3922‐3p on melanoma cells, we transfected miR‐542‐3p and miR‐3922‐3p mimics into *MLLT3* overexpressed A375 cells. As shown in Figure 3G,H, miR‐542‐3p and miR‐3922‐3p overexpression in A375 increased cell proliferation and invasion, which could be impaired by overexpression of *MLLT3*. Taken together, our above results demonstrated *MLLT3* was silenced by miR‐542‐3p and miR‐3922‐3p in melanoma cells.

### 2.4 MLLT3 Binds to HMGB1 and Inhibited its Nuclear Translocation

**Figure 4:**
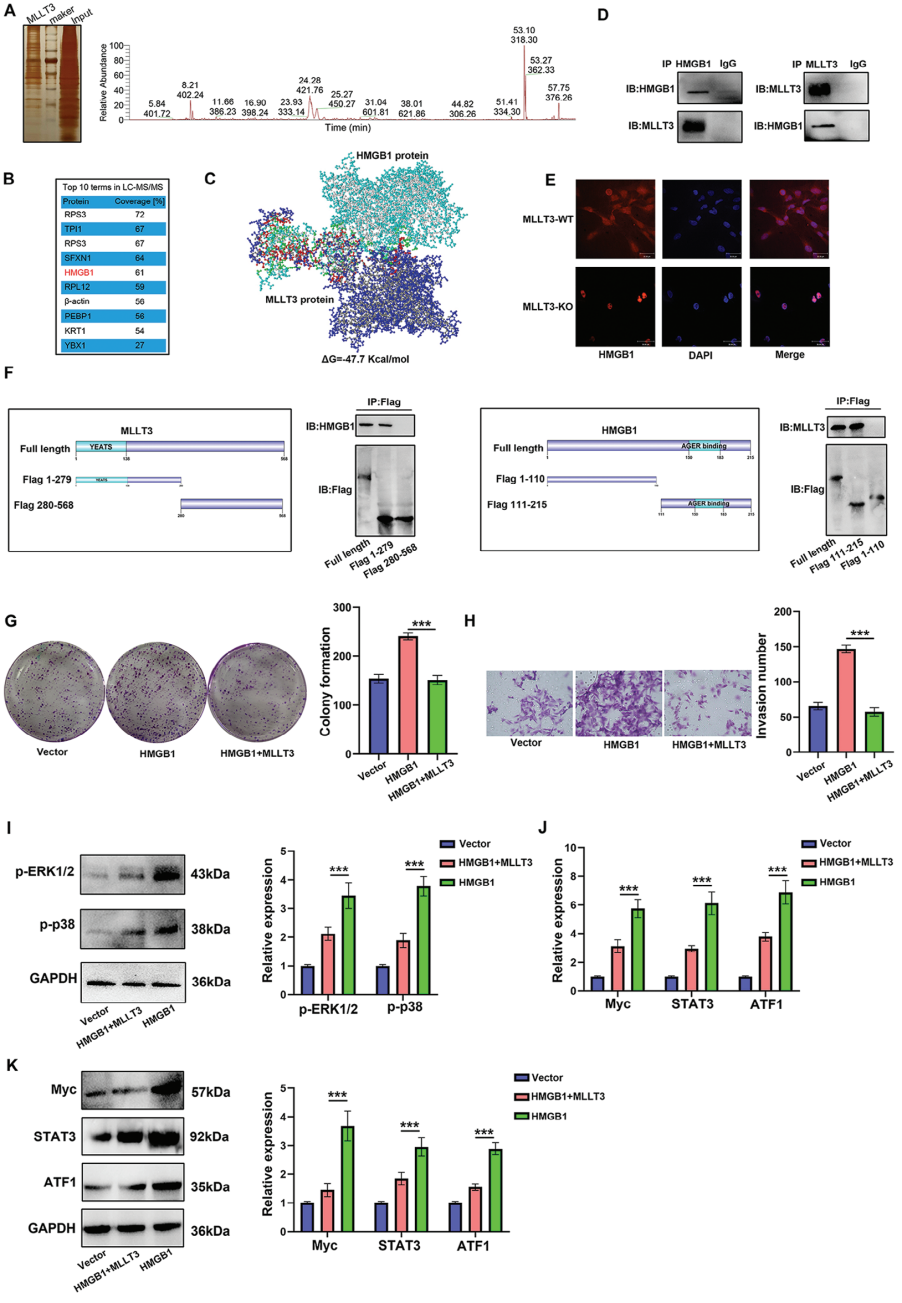
MLLT3 bound to HMGB1 and inhibited its nuclear translocation. A,B) Co‐immunoprecipitation (IP) followed by LC‐MS/MS was performed to seek the binding partners of MLLT3. C) The binding sites between MLLT3 and HMGB1 was predicted by molecular docking. D) The interaction between MLLT3 and HMGB1 was detected by endogenous Co‐IP. E) The localization of MLLT3 was measured by immunofluorescent staining. F) The binding sites between MLLT3 and HMGB1 was measured by exogenous Co‐IP. G) The effect of HMGB1 and MLLT3 on cell proliferation was detected by colony formation assay. H) The effect of HMGB1 and MLLT3 on cell invasion was measured by Transwell assay. I) Western blot analysis was used to detect MAPK signaling pathway by indicated antibodies. J) The expression of *Myc*, *STAT3* and *ATF1* was measured by qRT‐PCR after *HMGB1* and *MLLT3* co‐transfection. K) The expression of Myc, STAT3 and ATF1 was measured by western blot analysis after *HMGB1* and *MLLT3* co‐transfection. ***P < 0.001.

To detect the interaction proteins of MLLT3 in melanoma, we transfected HEK‐293T cells with the Flag‐MLLT3 plasmid and then purified the MLLT3‐ interacting proteins for LC‐MS/MS analysis. A series of peptides were detected, and one of the most abundant bands matched the peptide of HMGB1 (**Figure** **4**A,B). We also predicted the binding sites between MLLT3 and HMGB1 by molecular docking software (Figure 4C). In addition, the interaction between MLLT3 and HMGB1 was further confirmed by exogenous Co‐IP assays (Figure 4D). Next, by TCGA analysis, the expression of MLLT3 was not significantly correlated with HMGB1 in melanoma (Figure S4A, Supporting Information). In addition, the expression of HMGB1 was not significantly altered following MLLT3 overexpression and knockdown (Figure S4B, Supporting Information). However, the nuclear localization of HMGB1 was increased after MLLT3 overexpression, indicating MLLT3 inhibited its nuclear translocation (Figure 4E). To determine the interacting domain between MLLT3 and HMGB1, we made 2 truncations of the MLLT3 protein: the N‐terminus (Flag 1–279) and the C‐terminus (Flag 280–568). As shown in Figure 4F, the result showed the full length and N‐terminus could bind to HMGB1, suggesting that residues 1–279 might be necessary for interaction with HMGB1. For HMGB1, we also constructed two truncations: the N‐terminus (Flag 1–110) and the C‐terminus (Flag 111–215). The result of Co‐IP showed that C‐terminal domain (111‐215) of HMGB1 was essential for interaction with MLLT3. Next, we explored whether or not MLLT3 could inhibited cell proliferation and invasion via decreasing the nuclear translocation of HMGB1. As shown in Figure 4G,H, HMGB1 overexpression in A375 increased cell proliferation and invasion, which could be impaired by overexpression of MLLT3. It is well‐known that MAPK signaling pathway is a pivotal downstream target of HMGB1. We also detected the effect of MLLT3 on the activation of MAPK signaling pathway by western blot. The activation of p‐ERK1/2 and p‐P38 was significantly increased after HMGB1 overexpression, which could also be impaired by MLLT3 overexpression (Figure 4I). Furthermore, as shown in Figure 4J,K, the downstream targets of MAPK signaling pathway, such as *Myc*, *STAT3* and *ATF1*, were also regulated by *MLLT3* and *HMGB1*. Taken together, the above result demonstrated the C‐terminal domain of HMGB1 and N‐terminal domain of MLLT3 might be potential interaction domains. MLLT3 suppressed activation of MAPK signaling pathway via inhibiting the nuclear translocation of HMGB1.

### 2.5 *MAGEA1* was a Downstream Target of *MLLT3*

**Figure 5:**
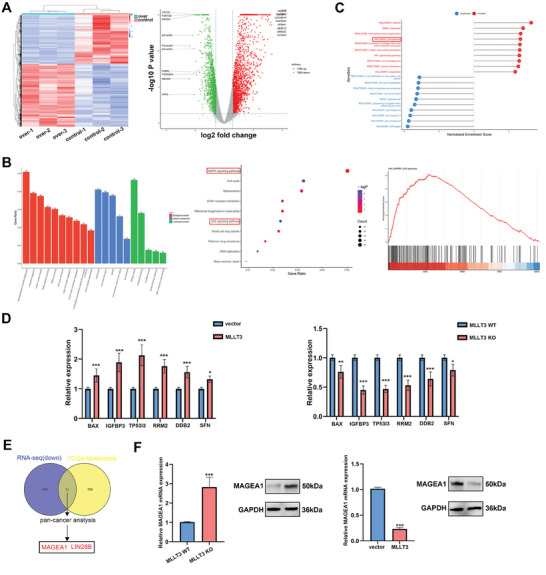
*MAGEA1* was a downstream target of *MLLT3*. A) RNA‐seq analysis of A375 cells with or without forced expression of *MLLT3*. B) The KEGG enrichment of RNA‐seq analysis. C) The GSEA enrichment of RNA‐seq analysis. D) The expression of P53 signaling pathway related genes was measured by qRT‐PCR after *MLLT3* overexpression in A375 cells. E) The overlapping genes of RNA‐seq analysis, TCGA Metastasis and Pan‐cancer analysis. F) The expression of *MAGEA1* was measured by qRT‐PCR and western blot after *MLLT3* overexpression and knockout. *P < 0.05, **P < 0.01, ***P < 0.001.

RNA sequencing (RNA‐seq) analysis was carried out in A375 cells following *MLLT3* overexpression in order to investigate the processes underpinning the impact of *MLLT3* on melanoma progression. As shown in **Figure** **5**A, we identified 1782 upregulated and 1383 downregulated genes (*P* value<0.05, foldchange>1.5) in comparison to control cells. Furthermore, Gene Set Enrichment Analysis (GSEA) and KEGG enrichment of RNA‐seq data demonstrated that *MLLT3* overexpression significantly regulated P53 signaling pathway and the MAPK signaling pathway (Figure 5B,C). To confirm the relationship between *MLLT3* and P53 signaling pathway‐related genes, qRT–PCR was performed after *MLLT3* overexpression. As shown in Figure 5D, the expression of P53 signaling pathway‐related genes, including *BAX*, *IGFBP3*, *TP53I3*, *RRM2*, *DDB2* and *SFN* was increased after *MLLT3* overexpression and decreased after *MLLT3* knockdown. Next, we analyzed the TCGA database to evaluate the RNA expression profile in order to better identify the underlying molecular targets of *MLLT3* in melanoma (Figure S5A,B, Supporting Information). By overlapping with TCGA RNA expression profile and pan‐cancer analysis, we found oncogene *MAGEA1* was a potential target of *MLLT3* (Figure 5E; Figure S5C, Supporting Information). As shown in Figure S5D (Supporting Information), the result of TCGA analysis indicated the expression of *MAGEA1* was up‐regulated in melanoma and correlated with tumor metastasis. In addition, we confirmed the effect of *MLLT3* on *MAGEA1* expression by qRT‐PCR and western blot assays. The result demonstrated the expression of *MAGEA1* was increased after *MLLT3* knockout and decreased after *MLLT3* overexpression (Figure 5F). To sum up, our result identified *MAGEA1* and P53 signaling pathway were downstream targets of *MLLT3* in melanoma.

### 2.6 MLLT3 Interacted with YBX1 and Inhibited the Recognition of *MAGEA1* m^5^C Modification

**Figure 6:**
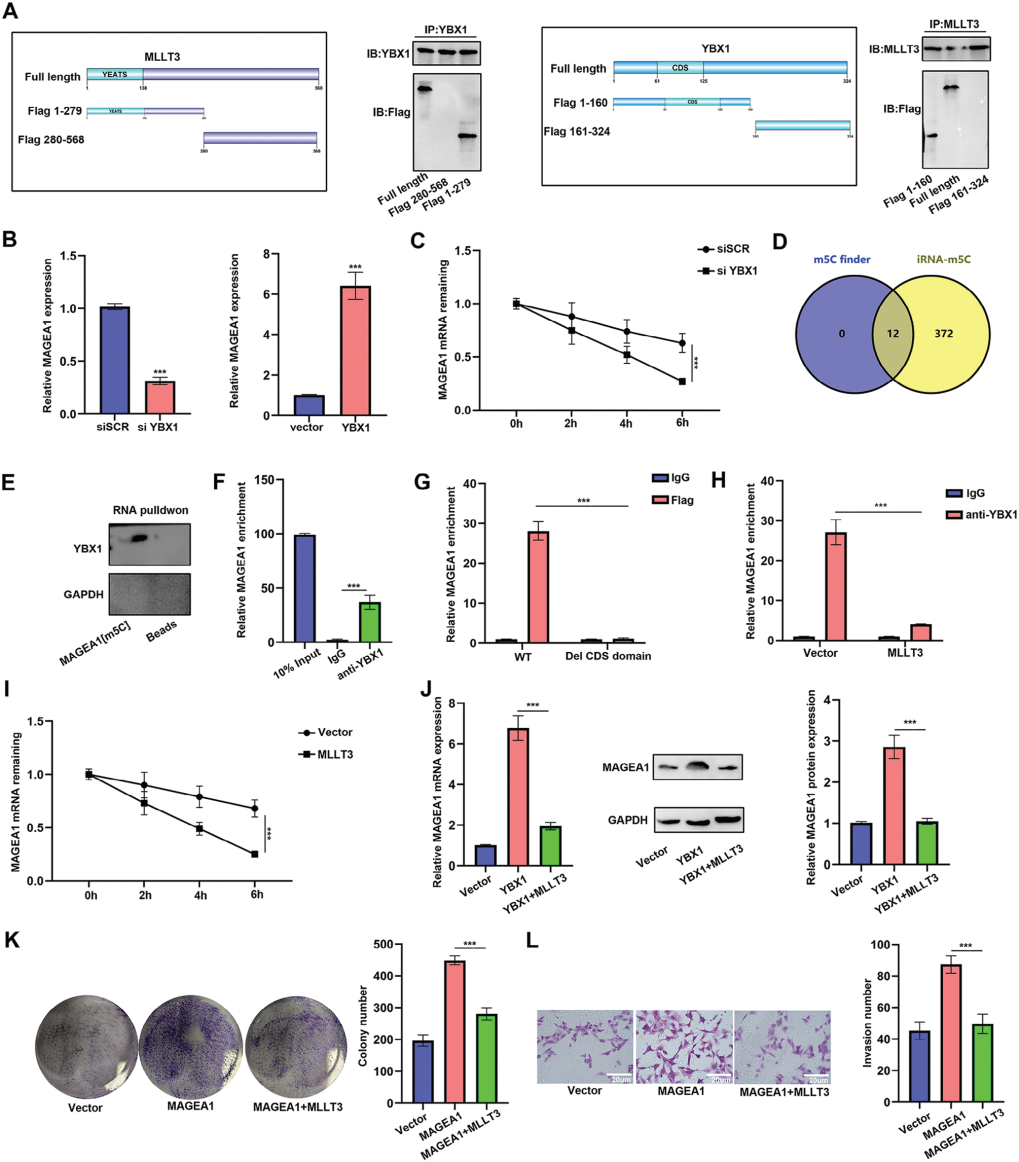
MLLT3 interacted with YBX1 and inhibited the recognition of *MAGEA1* m^5^C modification. A) The binding sites between MLLT3 and YBX1 was measured by exogenous Co‐IP. B) The expression of *MAGEA1* was detected by qRT‐PCR after *YBX1* knockdown and overexpression. C) The RNA stability assays were conducted using RT‐qPCR to measure the half‐life of *MAGEA1* mRNA in *YBX1* knockdown cells compared to control cells. D) The m^5^C modification sites of *MAGEA1* was predicted by m^5^C finder and iRNA‐m^5^C online tools. E) YBX1 immunoblot analysis of m^5^C‐MAGEA1 RNA pull‐downs in A375 cells. F) RIP assays verified the binding between YBX1 and *MAGEA1* mRNA. G) RIP assays evaluate the binding between full length or CSD domain deletion YBX1 and *MAGEA1* mRNA. H) The interaction between YBX1 and *MAGEA1* mRNA was measured by qRT‐PCR after *MLLT3* overexpression in A375 cells. I)The RNA stability assays were conducted using RT‐qPCR to measure the half‐life of *MAGEA1* mRNA after *MLLT3* overexpression. J) The expression of *MAGEA1* was measured by qRT‐PCR and western blot assay. K,L) The effect of *MAGEA*1 and *MLLT3* on cell proliferation and invasion was detected by colony formation and Transwell assay. ***P < 0.001.

Our previous result indicated that MLLT3 might interact with m^5^C reader YBX1 (Figure 4A,B). We next detected the expression of *YBX1* in melanoma by TCGA analysis. As shown in Figure S6A,B (Supporting Information), the expression of *YBX1* was significantly up‐regulated in melanoma and negatively correlated with the overall survival of patients. In addition, the expression of *MLLT3* was irrelevant with *YBX1* in melanoma (Figure S6C, Supporting Information), indicating *YBX1* was not regulated by *MLLT3*. Furthermore, the interaction between MLLT3 and YBX1 was validated by molecular docking and exogenous Co‐IP assays (Figure S6D,E, Supporting Information). The result of qRT‐PCR also confirmed the overexpression and knockdown of MLLT3 didn't change the expression of YBX1 (Figure S6F, Supporting Information). To determine the interacting domain between MLLT3 and YBX1, we also made 2 truncations of the YBX1 protein: the N‐terminus contained CDS domain (Flag 1–160) and the C‐terminus (Flag 161–324). As shown in **Figure** **6**A, the result of Co‐IP assay demonstrated the N‐terminal domain of MLLT3 and N‐terminal domain of YBX1 might be potential interaction domains. Next, we detected the effect of *YBX1* on the expression of *MAGEA1*. The knockdown of *YBX1* in melanoma cells led to a reduction in *MAGEA1* mRNA levels, while overexpression of *YBX1* significantly increased *MAGEA1* expression (Figure 6B). In addition, RNA stability assays also demonstrated that the knockdown of *YBX1* resulted in decreased stability of *MAGEA1* mRNA (Figure 6C). We also predicted the m^5^C modification sites of *MAGEA1* by m^5^C finder and iRNA‐m^5^C online software (Figure 6D; Figure S6G, Supporting Information). Next, the result of RNA pull‐down in conjunction with Western blot analysis provided additional validation that the m^5^C‐modified *MAGEA1* probe exhibits binding affinity toward YBX1, as opposed to the unmodified probe (Figure 6E). The interaction between *YBX1* and *MAGEA1* mRNA was further validated by RIP‐qPCR (Figure 6F). In addition, our findings also demonstrated the cold shock domain (CSD) of YBX1 is responsible for recognizing and binding to the m^5^C modification sites of *MAGEA1* (Figure 6G). Furthermore, the result of RIP assay demonstrated the interaction between YBX1 and *MAGEA1* mRNA was blocked by *MLLT3* overexpression (Figure 6H). Likewise, the RNA stability of *MAGEA1* was decreased after *MLLT3* overexpression (Figure 6I). Ultimately, the result of qRT‐PCR and western blot assays proved the effect of *YBX1* on *MAGEA1* could be reversed by *MLLT3* (Figure 6J). Cell functional experiments also demonstrated *MAGEA1* overexpression in A375 increased cell proliferation and invasion, which could be impaired by overexpression of *MLLT3* (Figure 6K,L). Taken together, our data indicated YBX1 was a m^5^C mediator that stabilizes *MAGEA1* mRNA through m^5^C modification and MLLT3 inhibited the recognition of *MAGEA1* m^5^C modification by blocking YBX1.

### 2.7 *MLLT3* Bound to Transcription Start Sites (TSSs) of P53 and Activated P53 Signaling Pathway

**Figure 7:**
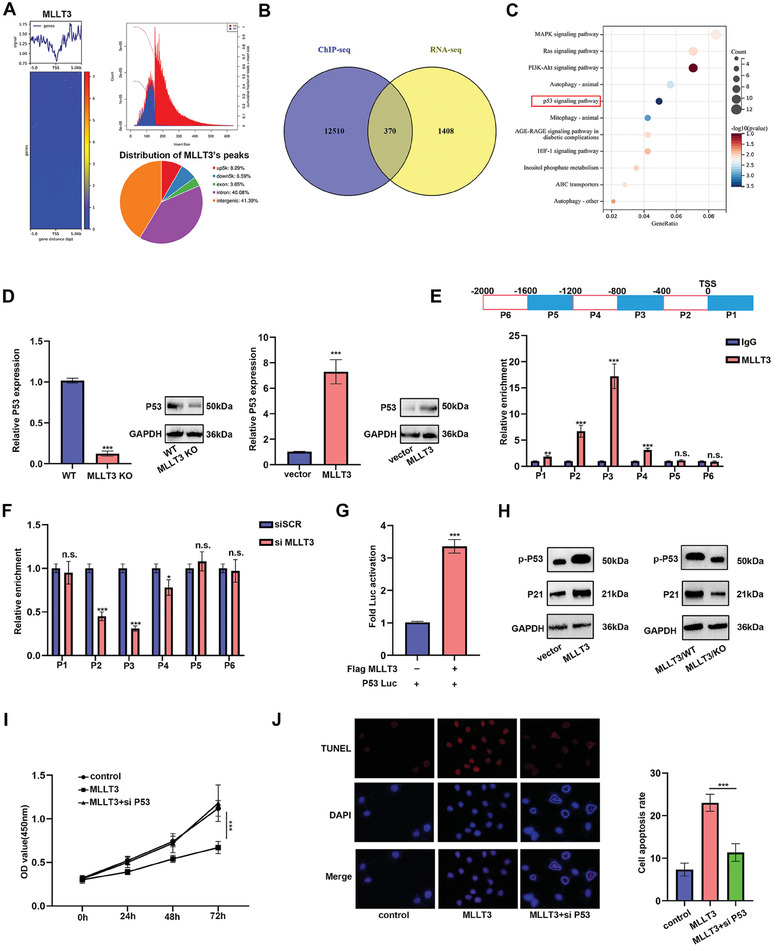
*MLLT3* bound to transcription start sites (TSSs) of P53 and activated P53 signaling pathway. A) The heatmap of ChIP‐seq data. B) A total of 370 genes were obtained after overlapping RNA‐seq and ChIP‐seq results. C) The KEGG enrichment of ChIP‐seq analysis. D) The expression of P53 was measured by qRT‐PCR and western blot after *MLLT3* overexpression and knockout. E) Increased *MLLT3* enrichment in the region of −1200–+400 bp from TSS of P53 promoter in A375 cells. F) The enrichment of *MLLT3* in P53 promoters was decreased after *MLLT3* knockdown. G) The interaction between *MLLT3* and P53 promoter was detected by luciferase reporter assay. H) The protein levels of p‐P53 and P21 was measured by western blot assay. I,J) The effect of *P53* and *MLLT3* on cell proliferation and apoptosis was detected by CCK‐8 and TUNEL assay. n.s. *P* > 0.05, *P < 0.05, **P < 0.01, ***P < 0.001.

To understand how *MLLT3* regulate melanoma progression, we assessed the *MLLT3* chromatin‐binding pattern in melanoma cells. Chromatin immunoprecipitation followed by ChIP‐seq showed *MLLT3* binding at 1329 sites, with strongest enrichment within 5 kb downstream of TSSs (**Figure** **7**A). To further detect the underlying molecular target of *MLLT3* in melanoma, we overlapped the result of ChIP‐seq and RNA‐seq data of *MLLT3* and performed KEGG enrichment. As shown in Figure 7B,C, P53 signaling pathway was considered as a potential target of *MLLT3*. The expression of qRT‐PCR and western blot assay also demonstrated the expression of P53 was primarily controlled by *MLLT3* at the level of transcription (Figure 7D). Next, the binding sites of *MLLT3* and P53 promoter was detected by ChIP‐qPCR. As shown in Figure 7E,F, the knockdown of *MLLT3* significantly decreased the enrichment of *MLLT3* in P53 promoter. In addition, the enrichment of *MLLT3* in the promoter of P53 was confirmed by luciferase reporter assay (Figure 7G). Moreover, we also detected the effect of *MLLT3* on the activation of P53 signaling pathway by western blot. The activation of p‐P53 and P21 was significantly increased after *MLLT3* overexpression and decreased after *MLLT3* knockout (Figure 7H). The result of CCK‐8 and Tunel assay indicated the proliferation of A375 cells was decreased and cell apoptosis was increased after *MLLT3* overexpression, which could be impaired by *P53* knockdown (Figure 7I,J).

Several studies reported the interaction between MAGE‐A proteins with P53 occluded the binding of P53 with P53‐responsive promoters, lead to the decreased P53‐ dependent transcription. Our result demonstrated that *MLLT3* inhibited the recognition of *MAGEA1* m^5^C modification by blocking *YBX1*. Thus we wondered whether or not *MLLT3* regulated P53 signaling via *MAGEA1*. As shown in Figure S7A,B (Supporting Information), the expression of P21 was negatively correlated with *MAGEA1* and regulated by *MAGEA1* in melanoma. In addition, the result of luciferase reporter assay demonstrated that *MAGEA1* inhibited the P53‐mediated up‐regulation of luciferase activity driven by P21 promoter, while *MLLT3* abolished the MAGEA1‐mediated inhibition of P21 promoter luciferase activity (Figure S7C, Supporting Information). Consisted with luciferase reporter assay, enforced expression of *MAGEA1* in A375 cells reduced P53‐mediated up‐regulation of endogenous P21 expression, whereas *MLLT3* reversed the reduction of endogenous P21 expression by targeting *MAGEA1* (Figure S7D, Supporting Information). Taken together, our results demonstrated *MLLT3* bound to transcription start sites (TSSs) of P53 and activated P53 signaling pathway. Meanwhile, *MLLT3* attenuated *MAGEA1* inhibited transcriptional activity of P53.

### 2.8 Single Cell Resolution of *MLLT3* Knockout

**Figure 8:**
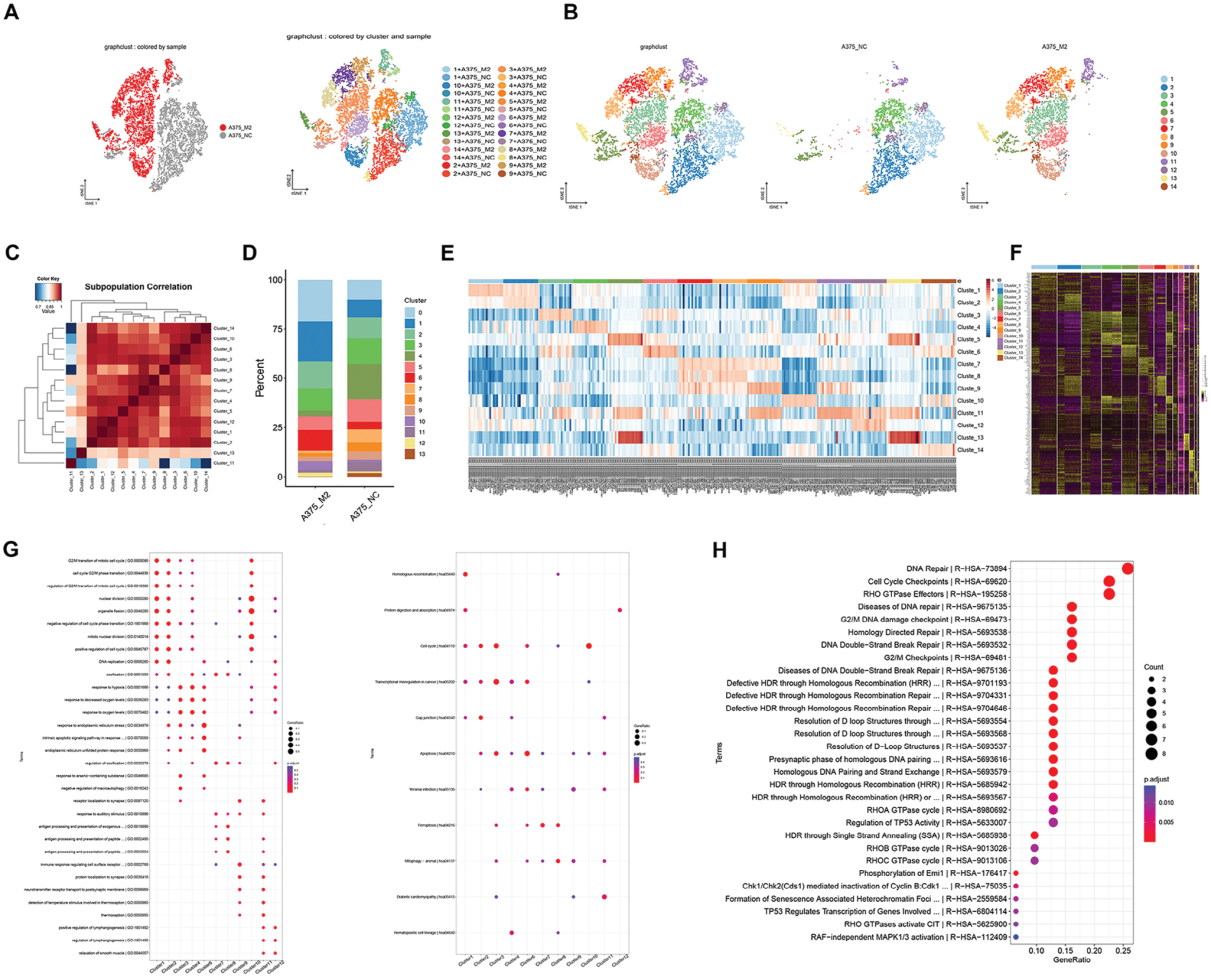
scRNA‐seq Shows *MLLT3* knockout‐Dependent biochemical mechanism. A,B) single cell RNA sequencing on the SK‐MEL‐2 cell line and identified 14 clusters. C) The correlation among cell subsets. D) The proportion of 0, 1, 4, 5, 6, 7, 8, and 10 clusters changed significantly. E) The expression patterns of top20 genes in different cell subgroups. F) The distribution of differential genes. G,H) GO, GSEA and KEGG analysis revealed that cell cycle G2/M phase transition(GO:0044839), positive regulation of cell cycle (GO:0045787), Cell cycle (hsa04110), Transcriptional misregulation in cancer(hsa05202), Regulation of TP53 Activity, TP53 Regulates Transcription of Genes Involved and RAF‐independent MAPK1/3 activation changed in melanoma cells.

Melanoma tumors and cell lines are known to be composed of a heterogeneous population, and it is unclear whether all cells within a melanoma are metastatic or if metastasisis restricted to a more aggressive subpopulation within tumors. To determinethe prevalence of *MLLT3*/KO cells, we performed single cell RNA sequencing (scRNAseq) on the SK‐MEL‐2 cell line (**Figure** **8**A) and identified 14 clusters representing a majority of cells that show a high degree of *MLLT3*/KO as illustrated in Figure 8B. In order to evaluate the results of cell clustering, we analyzed the correlation among cell subsets (Figure 8C), and analyzed the proportion of 14 cell clusters in cells (*MLLT3*/NC and *MLLT3*/KO cells), and found that the proportion of 0, 1, 4, 5, 6, 7, 8 and 10 clusters changed significantly (Figure 8D). The top20 genes of each subpopulation were analyzed by cluster analysis. The expression patterns of top20 genes in different cell subgroups can be obtained by cluster analysis, and whether genes in the same subgroup can be clustered into classes can be determined (Figure 8E). Next, the differential genes among the clusters were analyzed, and the distribution of differential genes was shown in the heat map (Figure 8F).

To uncover the biochemical mechanism by *MLLT3*, we performed GO, GSEA and KEGG analysis at single cell clusters differential gene level. As shown in Figure 8G,H, GO, GSEA and KEGG analysis revealed that cell cycle G2/M phase transition (GO:0044839); positive regulation of cell cycle (GO:0045787), Cell cycle (hsa04110), Transcriptional misregulation in cancer(hsa05202), Regulation of TP53 Activity, TP53 Regulates Transcription of Genes Involved and RAF‐independent MAPK1/3 activation, which provided evidences that *MLLT3* is involved in changes in melanoma cell proliferation, metastasis, and stemness at the single‐cell level.

### 2.9 *MLLT3* Inhibited Cell Proliferation and Invasion In Vivo

A subcutaneous tumor formation model was used to detect the function of *MLLT3* in vivo. As shown in Figure S8A (Supporting Information), the growth of tumors from the *MLLT3* overexpression group was significantly decreased compared to that of the NC group. The expression of PCNA (proliferation marker), CD133 and CD271 (stemness marker) was also decreased after *MLLT3* overexpression in vivo (Figure S8B, Supporting Information). Next, lung metastasis model was performed to measure the effect of *MLLT3* on cell invasion in vivo. As shown in Figure S8C (Supporting Information), compared to the NC group, lower lung metastases were observed in the *MLLT3* overexpression group. The expression of vimentin and snail (EMT maker) was also decreased after *MLTT3* overexpression in vivo (Figure S8D, Supporting Information).

## 3 Discussion

Malignant cancer is a serious threat to human health and life. More and more evidences show that cancer cells have high heterogeneity and plasticity.^[^ ^21^ ^]^ A small group of cells with unlimited proliferation potential in cancers that can reconstruct the occurrence of tumors are called cancer stem cells.^[^ ^22^ ^]^ Cancer stem cells maintain a relatively stable proportion in tumors through self‐renewal and differentiation.^[^ ^23^, ^24^ ^]^ In addition, cancer stem cells can migrate to the distal end through epithelial to mesenchymal transition and immune escape, resulting in cancer metastasis.^[^ ^25^ ^]^ Cancer stem cells are the fundamental factor of cancer occurrence, drug resistance, recurrence and metastasis, and also an important reason for the failure of cancer treatment.^[^ ^26^ ^]^ *MLLT3* can regulate the stemness of many cancers, and is a key stemness regulatory gene.^[^ ^6^, ^7^, ^8^, ^9^, ^10^, ^11^ ^]^ The role of *MLLT3* in the stemness and progression of melanoma cells is still unknown.

In this study, we explored the effect of *MLLT3* on melanoma through in vitro and in vivo experiments and clinical specimen analysis. As a result, we found a transcriptional regulatory gene that regulates the stemness of melanoma cells, and revealed that MLLT3 interacted with HMGB1 to inhibit its entry into the nucleus, and MLLT3 interacted with YBX1 to inhibit its reading of m^5^C of *MAGEA1*, thereby inhibiting the mRNA stability of *MAGEA1*, and directly transcribed P53 to inhibit the stemness, proliferation and metastasis of melanoma cells. The results of scRNA also confirm our conclusion. Overall, our results suggest that targeting *MLLT3* may provide the basis for new treatment strategies for melanoma.

Transcription factors are a group of proteins that bind to genes to regulate their expression.^[^ ^27^ ^]^ A large number of studies have suggested that dysregulated transcription factors are the main carcinogenic pathway of malignant cancers, which mainly lead to the occurrence and progress of cancer through the signal mechanism of proliferation, metastasis, reprogramming and cancer stemness.^[^ ^28^ ^]^ As a part of epigenetic content, the disorder of ncRNA can also promote the progress of cancer cells by regulating the stemness. Many miRNAs can directly interact with TF to regulate the proliferation, invasion, migration and stemness of cancer cells, and indirectly regulate the stemness of cancer cells through EMT.^[^ ^29^ ^]^ In the nucleus, HMGB1 is a DNA chaperone that maintains the structure and function of chromosomes.^[^ ^30^ ^]^ Many studies have shown that HMGB1 can increase the binding affinity between transcription factors and their homologous DNA sequences.^[^ ^31^ ^]^ The deletion of *HMGB1* reduces the efficiency of DNA repair, increases DNA damage and cell death, and weakens the response of cells to oxidative stress.^[^ ^32^ ^]^ *MEGEA1* is a member of MAGE‐A family and has strict tumor specificity. It is mainly expressed in malignant cancers or metastatic cancers.^[^ ^33^ ^]^RNA 5‐methylcytosine (m^5^C) is a common RNA modification in a variety of RNA species. YBX1, as a reader, regulates the apparent modification of m5C.^[^ ^34^ ^]^ In addition, the modification of m^5^C is closely related to the occurrence and development of cancer.^[^ ^35^, ^36^ ^]^ P53 is a powerful tumor suppressor, which can inhibit tumor growth in a variety of ways.^[^ ^37^ ^]^ Our results showed that MLLT3 interacted with HMGB1 to inhibit its entry into the nucleus. MLLT3 and YBX1 interact to prevent them from reading the m^5^C of *MAGEA1*, thus inhibiting the stability of *MAGEA1* mRNA. Direct transcription of p53 inhibits the stemness, proliferation and metastasis of melanoma cells. In addition, the potential mechanism of the interaction between miR‐542‐3p/miR‐3922‐3p and *MLLT3*. In vivo experiments also confirmed that overexpression of *MLLT3* significantly reduced the growth of melanoma, the metastasis rate, and the expression of PCNA and CD133. All the mechanisms by which MLLT3 regulates melanoma have not been discovered, so we believe that *MLLT3* plays an important role in regulating the stemness and progression of melanoma.

To support our hypothesis, scRNA showed significant changes in the cell subpopulations of melanoma cells with *MLLT3* knockout, activating the TP53 and MAPK pathways, providing evidence for the involvement of MLLT3 in melanoma cell proliferation, metastasis, and stem cell changes at the single‐cell level.

In this study, we identified *MLLT3* as a cancer suppressor gene of melanoma based on a variety of research methods. The experimental results and scRNA results echo each other, and the conclusion has high reliability. However, there are still some unsolved problems. First of all, we did not use genetically engineered mice as animal models, and we still do not know the occurrence and development of melanoma in *MLLT3* knockout mice. Second, we lack research on *MLLT3* targeted drugs, which is worth further exploration. Finally, the number of clinical specimens for melanoma validation is not sufficient, and more clinical samples still need to be collected for validation. In conclusion, our study suggests that targeting *MLLT3* may be an anti‐tumor strategy and a promising method for the treatment of melanoma.

We found that the transcription factor *MLLT3* is an inhibitory gene that regulates the stemness, occurrence and development of melanoma. It was revealed that MLLT3 interacted with HMGB1 to inhibit its entry into the nucleus, MLLT3 interacted with YBX1 to inhibit its reading of m^5^C of *MAGEA1*, thereby inhibiting the mRNA stability of *MAGEA1*, and directly transcribed p53 to inhibit the stemness, proliferation and metastasis of melanoma cells. We further explored the potential mechanism of the interaction between miR‐542‐3p/miR‐3922‐3p and *MLLT3*. Furthermore, the scRNA of melanoma cells with *MLLT3* knock‐out resulted in important changes in cell subsets, activating the TP53 and MAPK pathways and transforming into stem cells. In addition, overexpression of *MLLT3* significantly inhibited the stemness, proliferation, invasion and metastasis of melanoma cells. Therefore, *MLLT3* may be a promising therapeutic target for melanoma.

## 4 Experimental Section

### Constructing a Risk Score Model for Melanoma Stemness Related Genes

Download the expression profile data and clinical follow‐up information data of SKCM from the TCGA database, and download human cancer stemness related pathways from Molecular Signature Database v7.0 (MSigDB) and Gene Ontology (GO). A total of 398 genes related to cancer stemness were sorted out from 23 cancer stemness pathways. Next, the TCGA‐SKCM overall set (n = 450) will be divided into a training set (n = 300) and a testing set (n = 150) in a 2:1 ratio. In the training set, single factor Cox analysis will be used to identify 398 candidate genes, with a meaningful threshold set at p value<0.05. Further use the Least absolute shrinkage and selection operator (Lasso) method to screen variables and reduce the number of genes in the risk model. Finally, a multi factor Cox regression model was used to construct a Risk score model for melanoma stem cells.

### Patients and Tissues

Ten melanoma samples along with matched non‐tumorous tissues were collected from the First Hospital of China Medical University. All patients provided written informed consent, and the study received approval from the institution's Ethics Committee (Ref. No 2023.53). The melanoma tissues were snap‐frozen in liquid nitrogen immediately after surgical excision and stored at −80 °C.

### Immunohistochemistry

Immunohistochemical staining was conducted on paraffin‐embedded tissue sections from ten patient samples to analyze MLLT3 protein expression. Initially, the tissue slides were prepared by drying them at 37 °C overnight, followed by deparaffinization in xylene and rehydration through a series of graded ethanol solutions. To block endogenous peroxidase activity, the slides were immersed in 3% hydrogen peroxide for 10 min. Antigen retrieval was achieved using a citrate buffer solution (pH 6.0) heated in a microwave to enhance the accessibility of the target antigen. After cooling, the slides were incubated with an anti‐MLLT3 antibody (1:200 dilution, Abcam, Cambridge, UK, ab154492) overnight at 4 °C in a humidified chamber to ensure optimal binding. The following day, an immunohistochemistry SP kit (SP‐9000, ZSGB‐BIO, China) was used according to the manufacturer's protocol to apply the secondary antibody, followed by chromogenic detection with DAB to visualize positive staining. The slides were counterstained with hematoxylin, dehydrated through graded alcohol, cleared in xylene, and mounted with coverslips. Stained sections were observed under a microscope at 20× magnification, and MLLT3 protein expression was quantitatively analyzed using Image‐Pro Plus 6.0 software (Media Cybernetics, USA) to assess staining intensity and distribution within the tissue samples.

### Cell Culture

Melanoma cell lines (A375, HT‐144, WM‐115, and SK‐MEL‐2) were acquired from the Chinese Academy of Sciences and cultured in DMEM supplemented with 10% fetal bovine serum (FBS, Gibco) and 1% penicillin‐streptomycin. Melanocytes cell line PIG‐1 was acquired from the Chinese Academy of Sciences and cultured in 254 supplemented with Human Melanocyte Growth Supplement (HMGS, Thermo Scientific, S0025). The cultures were maintained in a humidified incubator at 37 °C with 5% CO_2_.

### CRISPR‐Cas9 and Gene Transfection

*MLLT3* knockout (KO) cells were generated using the CRISPR‐Cas9 system. Guide RNAs were designed using the CRISPOR website (http://crispor.tefor.net/) based on sequences from the UCSC genome database (http://genome.ucsc.edu/) and cloned into the lentiCRISPRv2 vector with a puromycin resistance marker. Control cells were transfected with an empty lentiCRISPRv2 vector (*MLLT3* wild type, WT). Guide RNA presence was confirmed by sequencing. LentiCRISPRv2 vectors, along with packaging plasmids pMD2.G and psPAX2, were transfected into 293FT cells to produce lentivirus. The virus was harvested 72 h post‐transfection, cleared of cell debris by centrifugation, and filtered through a 0.45µm filter. SK‐MEL‐2 cells were infected with the lentivirus and selected using 1µg mL^−1^ puromycin for seven days. Monoclonal cultures were established in 96‐well plates, expanded, and characterized by Sanger sequencing and Western blotting. For miR‐542‐3p and miR‐3922‐3p mimic transfection, A375 cells were seeded in six‐well plates at 50–60% confluence and transfected using Lipofectamine 2000, following the manufacturer's instructions. The *MLLT3* sgRNA sequences used are in Table S1 (Supporting Information).

### RNA Isolation and qRT‐PCR

Total RNA from tissues and cells was extracted using TRIzol reagent (Thermo Fisher Scientific, Inc) and reverse transcribed using the QuantiTect Reverse Transcription Kit (Qiagen). Quantitative real‐time PCR was performed using SYBR Green PCR mix (Takara), and gene expression was normalized to GAPDH or U6. Primer sequences are available in Table S1 (Supporting Information). In the qRT‐PCR experiment, the expression level of the target gene was quantified using the 2^‐ΔΔCt method. First, the Ct values of the target gene and the reference gene (GAPDH) were measured in both the experimental and control groups to calculate the ΔCt value (ΔCt = Ct_target gene – Ct_reference gene). Then, the ΔΔCt value was calculated (ΔΔCt = ΔCt_experimental group – ΔCt_control group), and the relative expression level of the target gene was determined using the 2^‐ΔΔCt formula. To compare gene expression differences between groups, an independent t‐test (Student's t‐test) was performed, setting a significance level of *P* < 0.05. All data were presented as mean ± standard deviation (mean ± SD), and the significance of the experimental results was evaluated based on the p‐value to determine whether there were statistical differences in gene expression between the groups.

### Cell Colony Formation, Migration and Invasion Assay

Cell colony formation, migration and invasion assay were performed as previously described.^[^ ^38^ ^]^

### RNA Pull‐Down Assay

By following the manufacturer's directions and treating the melanoma cell lysates with streptavidin‐coated magnetic beads (Sigma), the biotin‐coupled RNA complex was drawn down. To assess the enrichment of *MAGEA1* in the collected fractions, qRT‐PCR analysis was performed. Sangon Biotech provided both the control probe and the *MAGEA1* junction probe. Through the use of an anti‐YBX1 antibody in western blotting, the proteins inside the capture complex were identified. RNA immunoprecipitation (RIP) experiment, the input sample was first used as a control for normalization. Specifically, the expression levels of the target RNA in the RIP‐enriched samples and in the input samples were measured by qRT‐PCR, and the ΔCt value (ΔCt = Ct_target RNA – Ct_input) was calculated to account for sample differences. The relative RNA expression was then obtained using the 2^‐ΔCt method. To compare differences between the experimental and control groups, statistical analysis was performed using the independent t‐test (Student's t‐test), with a significance level set at *P* < 0.05. All data were presented as mean ± standard deviation (mean ± SD), and statistical significance was assessed based on the p‐value to determine whether there were significant differences between the groups.

### Co‐IP Assays

Co‐IP, the following specific procedures can be followed when using the Thermo Scientific co‐IP kit (NO. 88805) in an experiment. To put it succinctly, lysed cells were combined with non‐specific A/G agarose beads to create a supernatant. Following their removal, A/G agarose beads that were already coupled to HMGB1 and MLLT3 antibodies (Abcam) were treated with the supernatant. After the incubation period, the beads were cleaned, and Western blotting and SDS‐PAGE were used to elute and evaluate the bound proteins.

### ChIP‐Seq

The assay for chromatin immunoprecipitation (ChIP) was carried out in compliance with the manufacturer's instructions using a High‐Sensitivity ChIP Kit (Abcam). To improve DNA‐protein cross‐links, cells were, in essence, treated with 4% paraformaldehyde and then incubated with glycine for 20 min. Following cell lysing, DNA was sonicated to produce 400–800 bp chromatin fragments. Following sonication, the DNA fragments were subjected to an overnight immunoprecipitation process using Magnetic Protein A Beads coated with either rabbit nonimmune IgG (as a negative control) or MLLT3 antibody (Abcam), and send the final product for sequencing.

### RIP Assay

The Magna RIPTM RNA Binding Protein Immunoprecipitation Kit (Millipore) was used for the RNA immunoprecipitation (RIP) experiment in accordance with the manufacturer's instructions. To sum up, the cells were lysed in RIPA buffer that contained an RNase inhibitor and a protease inhibitor cocktail. Following this, the cells were treated at 4 °C for 6 h with buffer that contained magnetic beads conjugated with either immunoglobulin G (IgG) control or 5 µg human anti‐YBX1 antibody. To break down the proteins, proteinase K was then incubated with the beads. For additional research, isolated RNA was then subjected to RT‐PCR analysis.

### ScRNA‐Seq Sample Preparation and Data Analysis

In the scRNA‐seq comparisons of parental SK‐MEL‐2 and MLLT3/KO, sub‐confluent (≈40% confluent) cells were trypsinized. These cells were then prepared following the 10X Genomics sample preparation guidelines and counted using a hemocytometer. The cells were suspended in 0.04% BSA/PBS, and libraries were created using 10X Genomics v3 Chemistry, aiming for 5000 cells per condition. Sequencing was performed on an Illumina HiSeq 4000 with 150 base pair, paired end reads. All libraries exceeded the 10X Genomics recommended minimum of 20 000 average reads per cell. Data processing for scRNA‐Seq analysis using cell ranger and Seurat.

### Western Blot Assay

RIPA Lysis with Protease/Phosphatase Inhibitor Cocktail (Abcam) was used to lyse the cells. Following their separation on a 10% SDS‐polyacrylamide gel, the extracted proteins were transported to immobilon‐P membranes from Merck Millipore. After blocking the membrane for an hour at room temperature using 5% nonfat dried milk in PBS containing 0.1% Tween 20, the membrane was treated with primary antibodies for an additional night at 4 °C. The following day, the membranes were incubated with HRP‐conjugated secondary antibody (1:1000, Santa Cruz Biotech) for 2 h at room temperature after being cleaned three times with Tris‐buffered saline Tween. Ultimately, an Amersham Biosciences ECL Western blotting kit was used to detect every band. GAPDH served as the internal point of reference. The following antibodies were used: anti‐MLLT3 (Cell Signaling Technology, Danvers, MA, USA), anti‐E‐cadherin (Proteintech, Wuhan, China), and anti‐Vimentin (Sigma, St. Louis, MO, USA), anti‐Snail, anti‐CD133, anti‐GAPDH, anti‐HMGB1, anti‐p‐ERK1/2, anti‐p‐p38, anti‐MEGEA1, anti‐YBX1, anti‐P53, anti‐p‐P53, anti‐P21, anti‐PCNA (Abcam, Cambridge MA, USA). To quantitatively analyze the experimental data, the grayscale values of the target protein band and the GAPDH internal control band were first measured using ImageJ software. The grayscale ratio of the target protein to GAPDH was then calculated to correct for potential loading differences between samples. Subsequently, statistical analysis was performed by comparing the grayscale ratios of the experimental group and the control group using an independent t‐test. All statistical analyses were conducted using GraphPad Prism software, with a significance level set at *P* < 0.05. Finally, based on the statistical results, the differences were assessed in target protein expression between the experimental and control groups and drew conclusions accordingly.

### Xenografts Assays

The National Institutes of Health Guide for the Care and Use of Laboratory Animals was followed, and the Animal Center of the Institute of peking university shenzhen hospital authorized the animal experiments (No. 2023–861). Our study examined male and female animals, and similar findings were reported for both sexes. A375 cells that stably expressed MLLT3 or an empty vector were subcutaneously implanted into 4‐week‐old nude mice (BALB/c‐nu, HFK Bioscience, Beijing, China). Every seven days, the tumor's size was measured. A lung metastasis model was utilized to quantify cell metastasis in vivo. In summary, 0.1 mL of phosphate‐buffer saline containing 1×10^6^ A375 cells (either MLLT3 or empty vector) was introduced into the tail vein of naked mice. The animals were put to sleep after four weeks of feeding, and the lung tissues were taken out for additional processing. After 4 weeks of the experiment, the xenograft tumors were removed and weighed, and the mice were put to sleep with an intraperitoneal injection of 100 mg kg^−1^ pentobarbital sodium (Sigma, St. Louis, MO, USA).

### Dual‐Luciferase Reporter Assay

MLLT3 wild‐type (WT) and mutant sequences, with or without the miR‐542‐3p /miR‐3922‐3p and P53 binding site, were cloned and inserted into pGL3 luciferase plasmids and co‐transfected with miR‐542‐3p /miR‐3922‐3p and P53 into HEK‐293 cells. Twenty‐four hours after transfection, the cells were collected and lysed. Luciferase activities were evaluated using a dual‐luciferase reporter assay system (Promega, Madison, USA). The intensities of the firefly and Renilla luciferases were recorded and measured. The experimental data were analyzed using GraphPad Prism software, and differences between groups were compared using the independent t‐test (Student's t‐test). A significance level of *P* < 0.05 was set for statistical analysis, and all data were presented as mean ± standard deviation (mean ± SD). The significance of the experimental results was evaluated based on the p‐value to determine whether there were significant differences in transcriptional activity between the groups.

### Statistical Analysis

Software for statistical analysis was SPSS 18.0, and mapping was done with Graphpad Prism 9. As said, the data were shown as the mean ± SD. An unpaired, two‐tailed t‐test was used to compare the two groups; a p‐value of less than 0.05 was deemed statistically significant.

## Conflict of Interest

The authors declare no conflict of interest.

## Author Contributions

Y.L. and H.L. contributed equally to the work. Y.L. and B.S. designed the experiments. Y.L., H.L., J.L., and C.F. conducted experiments and obtained the data. Y.L. analyzed the data. B.S. supervised the research. Y.L. and B.S. wrote the manuscript. B.J., B.C., Y.Z., and B.Y. helped with the experimental design and edited the manuscript. All authors reviewed the manuscript and agreed to the final version.

## Supporting information



Supporting Information

Supporting Table 1

## Data Availability

Gene expression data were deposited in the Genome Sequence Archive (GSA) in National Genomics Data Center (NGDC) of the Chinese Academy of Sciences (https://ngdc.cncb.ac.cn/gsa; GSA accession: CRA017514). All data used for figures are available in the Supporting Data Values file. All data can be requested from the corresponding author.
