# Supplementary material for: MLLT3 Regulates Melanoma Stemness and Progression by Inhibiting HMGB1 Nuclear Entry and MAGEA1 M5C Modification
Source: Adv Sci (Weinh). 2024 Dec 24;12(10):2408529. doi: 10.1002/advs.202408529 (PMC11904942; doi:10.1002/advs.202408529)
Supplement: Supplementary file 1 — Supporting Information [file ADVS-12-2408529-s002.docx]

**MLLT3 regulates melanoma stemness and progression by inhibiting HMGB1 nuclear entry and MAGEA1 m^5^C modification**

Yaling Li^1,2,3#^*, Hong Liu^4#^, Jingyi Li^1,3^, Chang Fu^1,3^, Bin Jiang^2^, Bancheng Chen^2^, Yanfen Zou^2^, Bo Yu^2^*, Bing Song^1,3^*

1 Institute of Biomedical and Health Engineering, Shen Zhen Institutes of Advanced Technology, Chinese Academy of Science, Shenzhen 518055, Guangdong, China

2 Department of Dermatology, Institute of Dermatology, Peking University Shenzhen Hospital, Shenzhen Peking University-The Hong Kong University of Science and Technology Medical Center, Shenzhen 518036, Guangdong, China.

3 Department of Dermatology, the First Hospital of China Medical University, Shenyang 110001, Liaoning, China

4 Department of Otorhinolaryngology, Xiang'an Hospital of Xiamen University, Xiamen 361000, Fujian, China.

#Contributed equally to the paper. *Correspondence: Bing Song, Email: [bing.song@siat.ac.cn](mailto:bing.song@siat.ac.cn); Bo Yu, Email: [yubomd@163.com](mailto:yubomd@163.com); Yaling Li, Email: allenli666@foxmail.com.

Supplemental materials:

• Supplementary Figures 1-8

• Supplemental Table 1

**
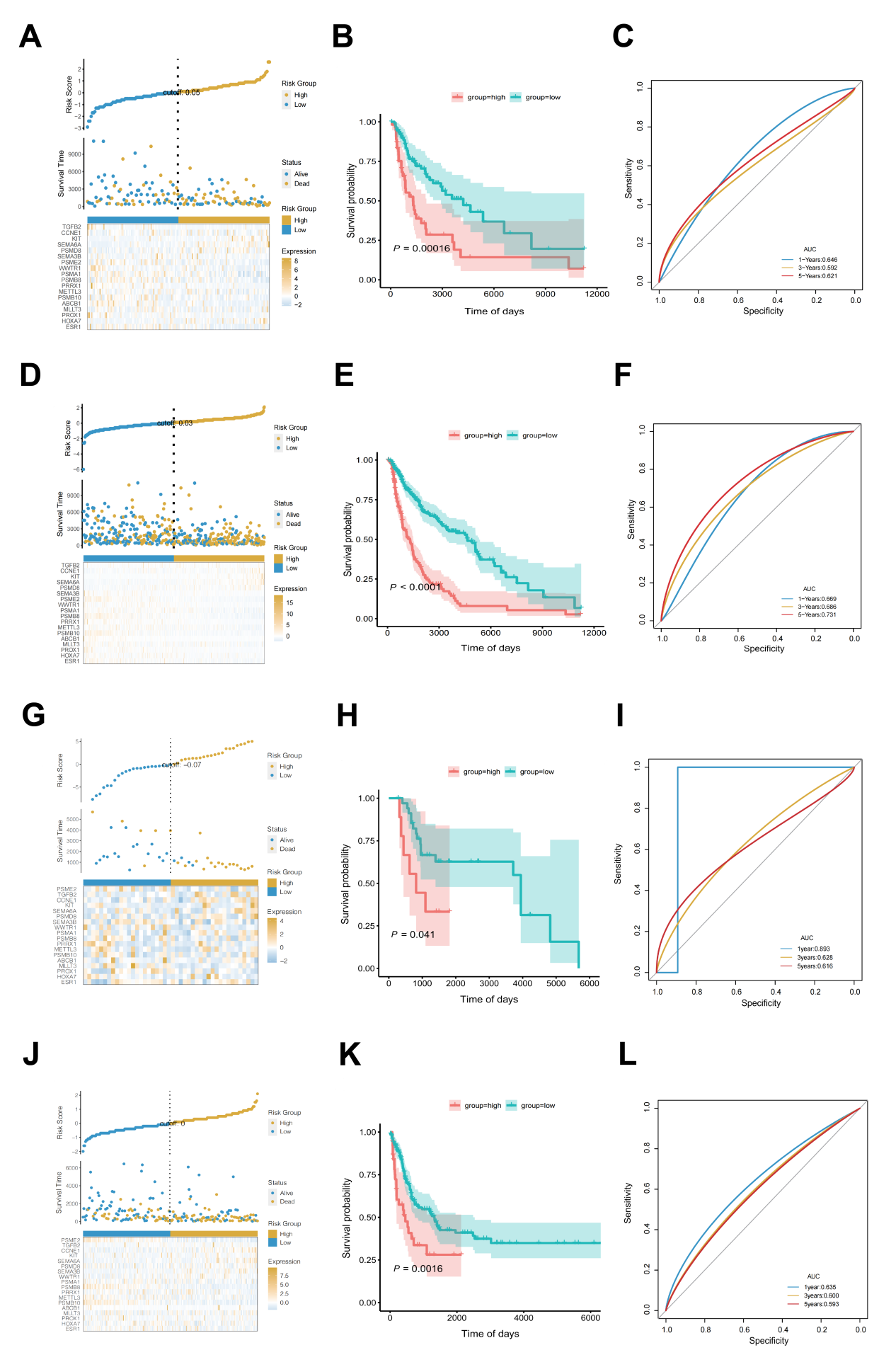
**

**Figure S1: Verify the risk score model.** (**A**) Distribution diagram of risk scoring model of test set. (**B**) The survival curve of the test set risk score model. (**C**) ROC curve of test set risk scoring model. (**D**) The distribution map of the integrated risk scoring model. (**E**) The survival curve of the whole set risk scoring model. (**F**) ROC curve of the whole set risk scoring model. (**G**) The distribution diagram of risk scoring model of GSE19234 dataset. (**H**) The survival curve of GSE19234 data set risk scoring model. (**I**) ROC curve of GSE19234 dataset risk scoring model. (**J**) The distribution diagram of risk scoring model of GSE65904 dataset. (**K**) The survival curve of GSE65904 data set risk scoring model. (**L**) ROC curve of GSE65904 dataset risk scoring model.


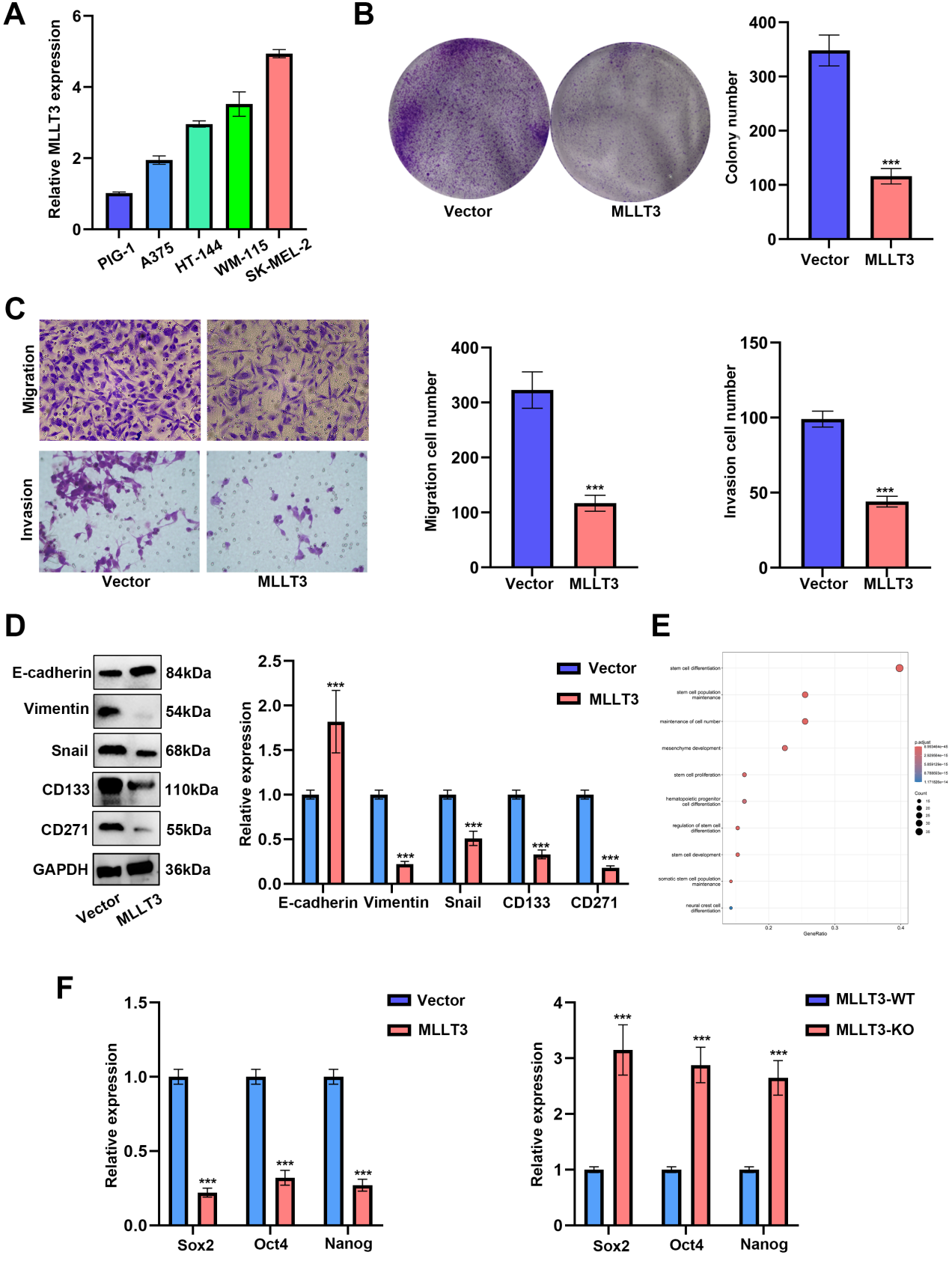


**Figure S2: *MLLT3* overexpression promoted proliferation, metastasis, invasion, and stemness of melanoma cells.** (**A**) The expression *MLLT3* was measured by qRT-PCR in melanoma cells and melanocytes. (**B**) The proliferation of A375 cells was detected by colony formation after *MLLT3* overexpression. (**C**) Transwell migration assay and invasion assay were performed after *MLLT3* overexpression. (**D**) The protein levels of EMT and stemness markers were measured by western blot after *MLLT3* ovexoression. (**E**) GO analysis based on MLLT3 as the grouping variable. (**F**) The expression of stemness-related genes were evaluated in melanoma stem cells after MLLT3 overexpression or knockout. ****P* < 0.001.


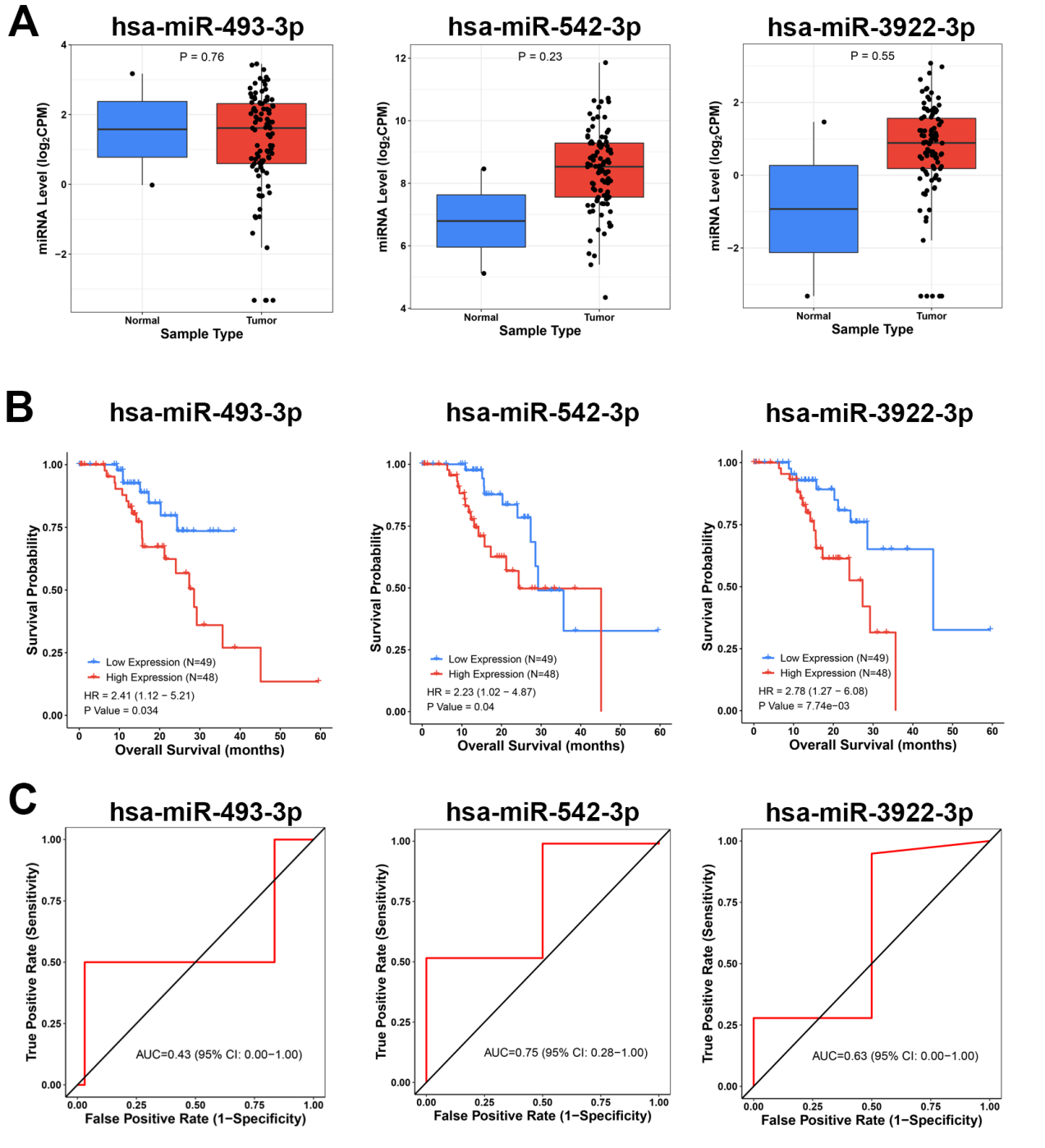


**Figure S3: The expression, survival analysis and ROC curve of miR-493-3p, miR-542-3p and miR-3922-3p.** (**A**) The expression of miR-493-3p, miR-542-3p, miR-3922-3p was analyzed by TCGA in melanoma. (**B**) The survival analysis of miR-493-3p, miR-542-3p, miR-3922-3p was performed by TCGA data in melanoma. (**C**) The ROC curve of miR-493-3p, miR-542-3p, miR-3922-3p based on TCGA data.


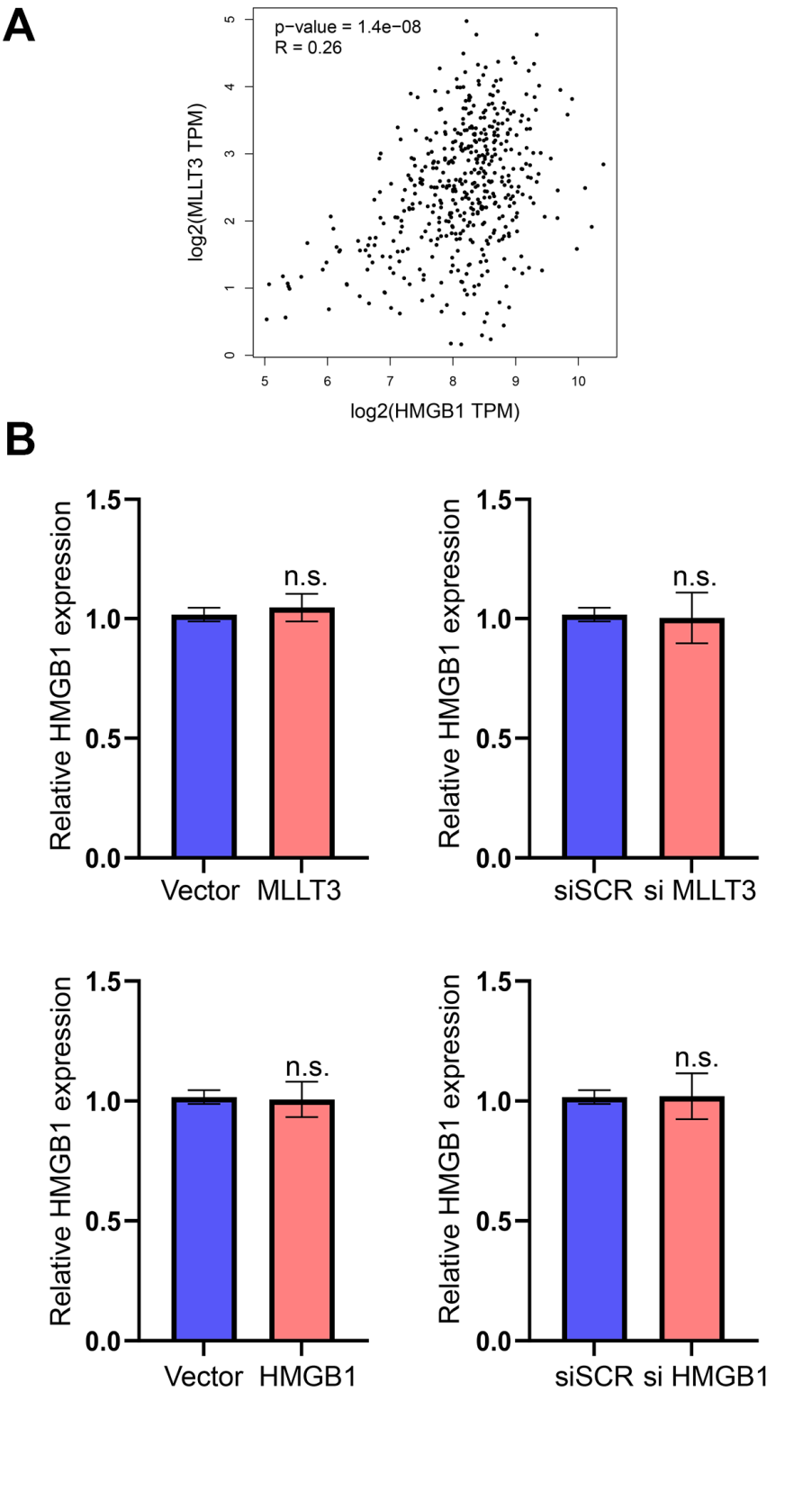


**Figure S4:** **The expression of *MLLT3* was not significantly correlated with *HMGB1* in melanoma.** (**A**) The correlation between *MLLT3* and *HMGB1* based on TCGA data. (**B**) The expression of *MLLT3* and *HMGB1* was measured by qRT-PCR after *HMGB1*/*MLLT3* overexpression or knockdown. n.s. *P* > 0.05.

**
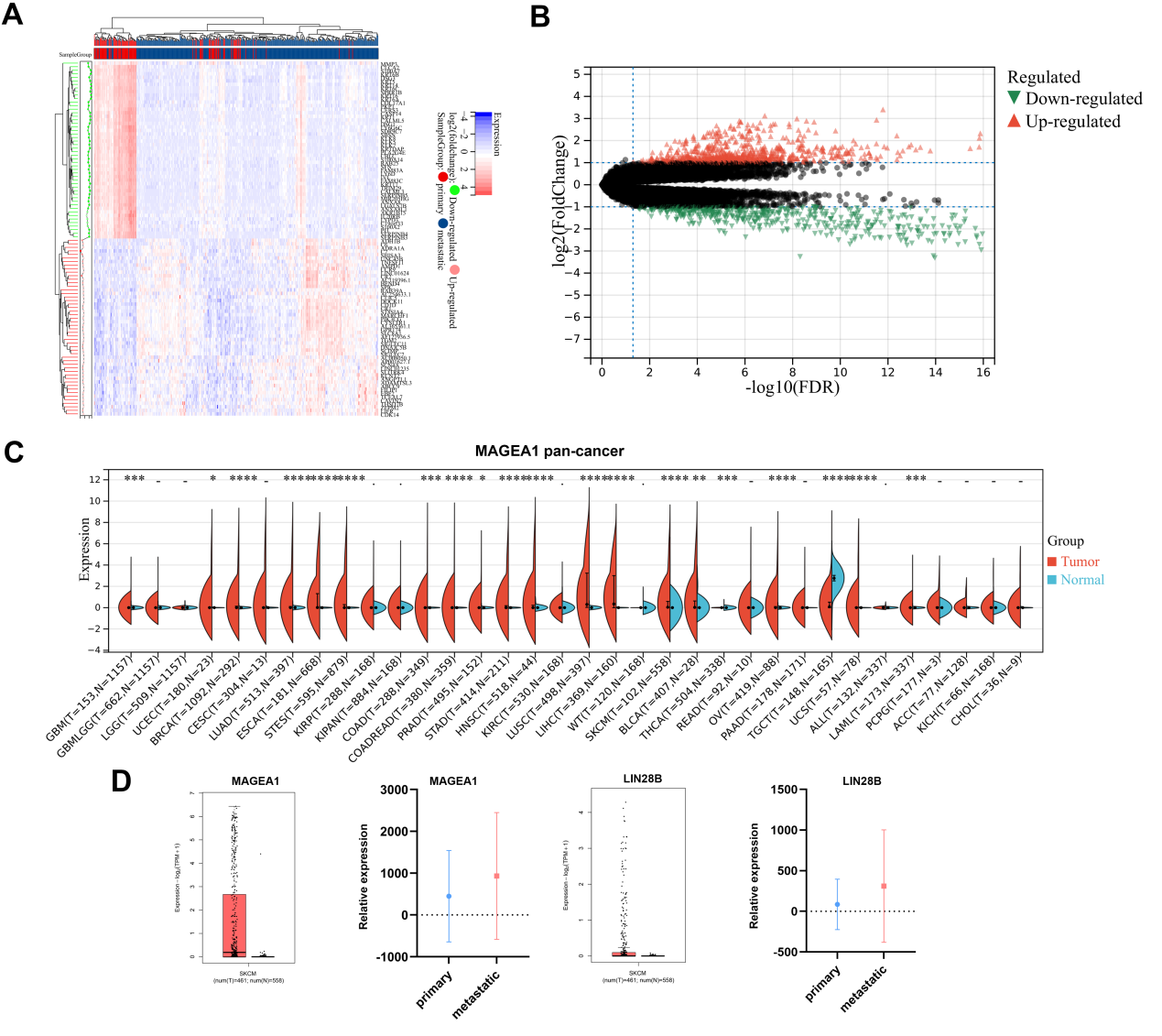
**

**Figure S5: *MAGEA1* was a potential target of *MLLT3*.** (**A-B**) The heatmap and volcano map of TCGA analysis. (**C**) The pan-cancer analysis of *MAGEA1*. (**D**) The expression of *MAGEA1* and *LIN28B* based on TCGA in melanoma. **P* < 0.05, ***P* < 0.01, ****P* < 0.001, *****P* < 0.0001.


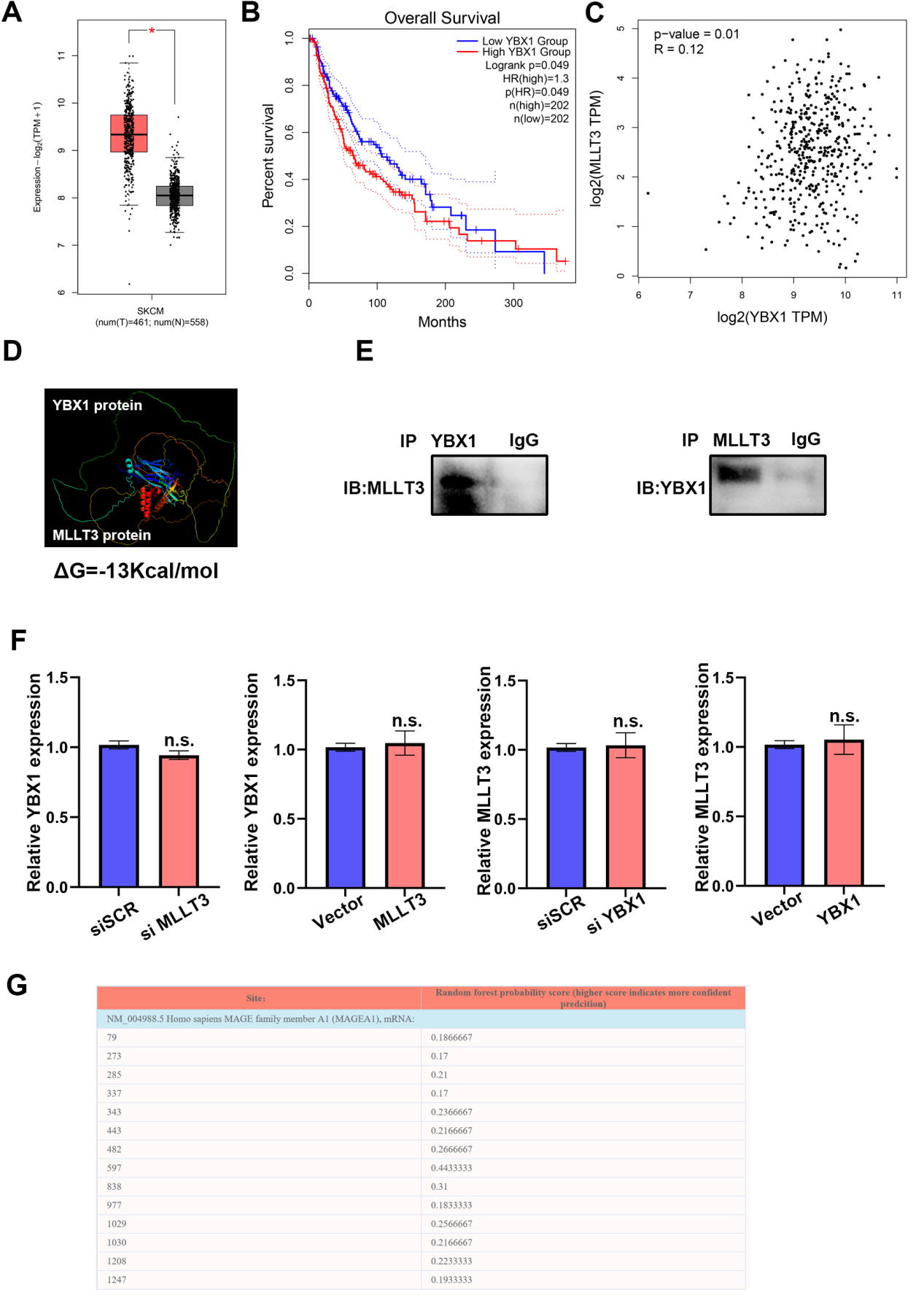


**Figure S6: Interaction between MLLT3 and YBX1.** (**A**) The expression analysis of *YBX1* was performed by TCGA in melanoma. (**B**) The survival analysis of *YBX1* was performed by TCGA in melanoma. (**C**) The correlation between *MLLT3* and *YBX1* based on TCGA data. (**D**) The binding sites between MLLT3 and YBX1 was predicted by molecular docking. (**E**) The interaction between MLLT3 and YBX1 was detected by endogenous Co-IP. (**F**) The expression of *MLLT3* and *YBX1* was measured by qRT-PCR after *HMGB1*/*YBX1* overexpression or knockdown. (**G**) The m^5^C modification sites of *MAGEA1* was predicted by iRNA-m^5^C online tools. n.s. *P* > 0.05, **P* < 0.05.


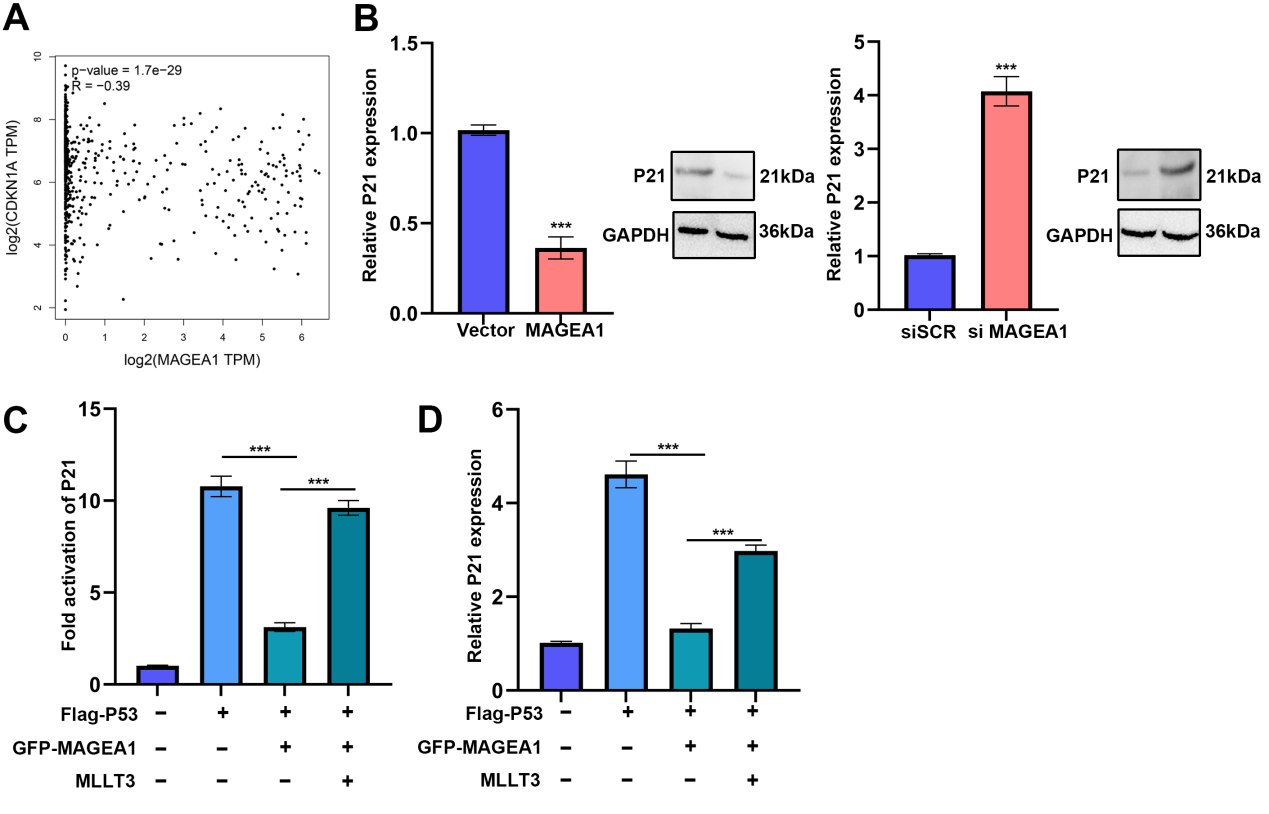


**Figure S7:** **The correlation between P21 and MAGEA1.** (**A**) The correlation between *P21* and *MAGEA1* based on TCGA data. (**B**) The expression of P21 was measured by qRT-PCR and western blot assay after *MAGEA1* overexpression and knockdown. (**C**) Luciferase reporter assays for luciferase activity of luc-p21 promoter in A375 cells co-transfected with combination of different plasmids. (**D**) The expression of *P21* in A375 cells co-transfected with combination of different plasmids was examined by qRT-PCR. ****P* < 0.001.


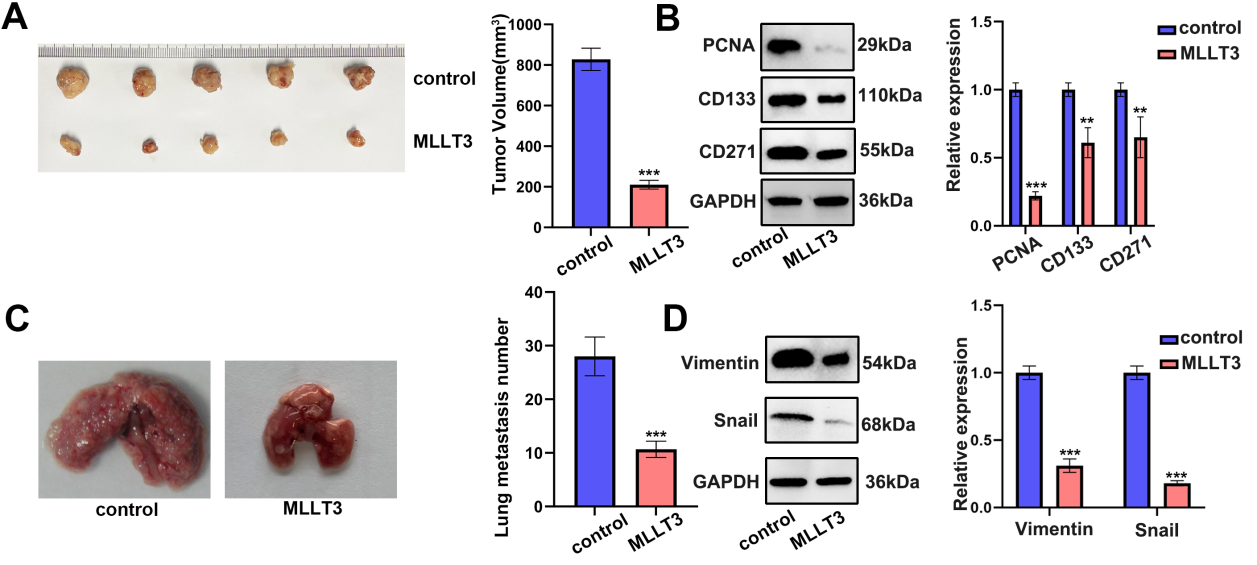


**Figure S8: *MLLT3* inhibited cell proliferation and invasion in vivo.** (**A**) Pictures of the subcutaneous transplanted tumor. (**B**) The expression of PCNA, CD133 and CD271 was measured by western blot assay. (**C**) Pictures of the lung metastasis model. (**D**) The expression of Vimentin and Snail was measured by western blot assay. ***P* < 0.01, ****P* < 0.001.

**Table S1. Gene sequence information.**

| Gene | Sequence |
| --- | --- |
| MLLT3 | sgRNA1-AGCTTTCCTAGGCCAAAAAG |
|  | sgRNA2-CAGCGGAGGTGATTCACTGG |
|  | sgRNA3-GGATCCCAATGATTCAGATG |
|  | sgRNA4-GTACGAACACCATCCAGTCG |
| MLLT3-F | CAGATGAAGTGGAGGATAACGAC |
| MLLT3-R | TGGTGGAGGTTCGTGATGTAGG |
| E Cadherin (CDH1)-F | GCCTCCTGAAAAGAGAGTGGAAG |
| E Cadherin (CDH1)-R | TGGCAGTGTCTCTCCAAATCCG |
| Vimentin (VIM)-F | AGGCAAAGCAGGAGTCCACTGA |
| Vimentin (VIM)-R | ATCTGGCGTTCCAGGGACTCAT |
| SNAIL (SNAI1)-F | TGCCCTCAAGATGCACATCCGA |
| SNAIL (SNAI1)-R | GGGACAGGAGAAGGGCTTCTC |
| MAGEA1-F | CACTACCAAGGACAAGGCGTTC |
| MAGEA1-R | CAACGCCTCTTTGGTCTCCTTG |
| MLLT3-F | GCACCTCTTGTATCCTGGAGTC |
| MLLT3-R | GACACTCTCCAGCATTTCTGCC |
| YBX1-F | GCAGGAGAACAAGGTAGACCAG |
| YBX1-R | CTTCATTGCCGTCCTCTCTAGG |
| P53-F | CCTCAGCATCTTATCCGAGTGG |
| P53-R | TGGATGGTGGTACAGTCAGAGC |
| PCNA-F | CAAGTAATGTCGATAAAGAGGAGG |
| PCNA-R | GTGTCACCGTTGAAGAGAGTGG |
| GAPDH-F | GTCTCCTCTGACTTCAACAGCG |
| GAPDH-R | ACCACCCTGTTGCTGTAGCCAA |
| U6-F | CTCGCTTCGGCAGCACAT |
| U6-R | TTTGCGTGTCATCCTTGCG |

**Table S2. Identification of candidate genes by univariate Cox analysis.**
